# Conformational stability of SARS-CoV-2 glycoprotein spike variants

**DOI:** 10.1016/j.isci.2022.105696

**Published:** 2022-11-30

**Authors:** Hiam R.S. Arruda, Tulio M. Lima, Renata G.F. Alvim, Fernanda B.A. Victorio, Daniel P.B. Abreu, Federico F. Marsili, Karen D. Cruz, Mayra A. Marques, Patricia Sosa-Acosta, Mauricio Quinones-Vega, Jéssica de S. Guedes, Fábio C.S. Nogueira, Jerson L. Silva, Leda R. Castilho, Guilherme A.P. de Oliveira

**Affiliations:** 1 Institute of Medical Biochemistry Leopoldo de Meis, National Institute of Science and Technology for Structural Biology and Bioimaging, National Center of Nuclear Magnetic Resonance Jiri Jonas, Federal University of Rio de Janeiro, Rio de Janeiro, RJ 21941-902, Brazil; 2 Cell Culture Engineering Laboratory, COPPE, Federal University of Rio de Janeiro (UFRJ), Rio de Janeiro, RJ 21941-598, Brazil; 3 EPQB Program, School of Chemistry, Federal University of Rio de Janeiro (UFRJ), Rio de Janeiro, RJ 21941-598 Brazil; 4 Biochemistry Program, Institute of Chemistry, Federal University of Rio de Janeiro (UFRJ), Rio de Janeiro, RJ 21941-909, Brazil; 5 Laboratory of Proteomics (LabProt), LADETEC, Institute of Chemistry, Federal University of Rio de Janeiro, Rio de Janeiro, RJ 21941-598, Brazil

**Keywords:** Biochemistry, Virology, Structural biology, Protein structure aspects

## Abstract

The severe acute respiratory syndrome spread worldwide, causing a pandemic. SARS-CoV-2 mutations have arisen in the spike, a glycoprotein at the viral envelope and an antigenic candidate for vaccines against COVID-19. Here, we present comparative data of the glycosylated full-length ancestral and D614G spike together with three other transmissible strains classified by the World Health Organization as variants of concern: beta, gamma, and delta. By showing that D614G has less hydrophobic surface exposure and trimer persistence, we place D614G with features that support a model of temporary fitness advantage for virus spillover. Furthermore, during the SARS-CoV-2 adaptation, the spike accumulates alterations leading to less structural stability for some variants. The decreased trimer stability of the ancestral and gamma and the presence of D614G uncoupled conformations mean higher ACE-2 affinities compared to the beta and delta strains. Mapping the energetics and flexibility of variants is necessary to improve vaccine development.

## Highlights

• Comparative biochemical data of the ancestral, D614G, beta, gamma, and delta spikes
• D614G has less hydrophobic exposure and trimer persistence at mid temperatures
• The ancestral and gamma spikes have similar glycan abundance
• SARS-CoV-2 evolution alters the spike stability for some variants

## Graphical abstract

**Figure:**
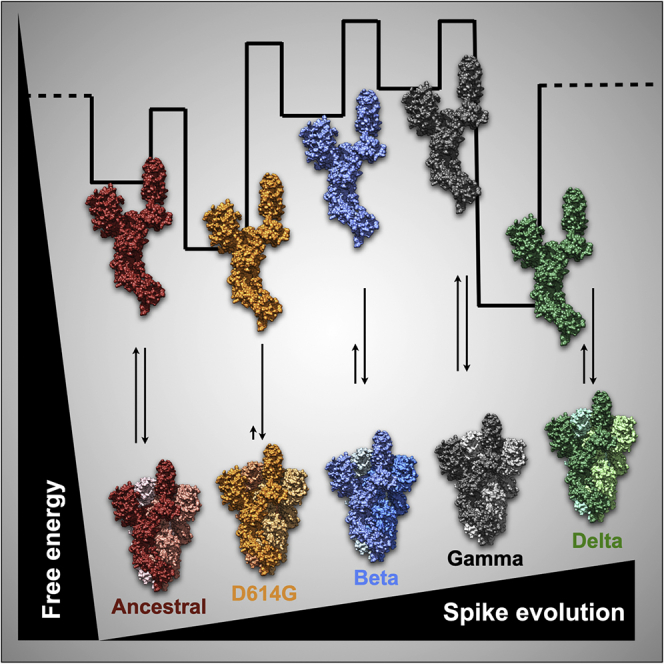

## Introduction

Pathogenic coronaviruses (CoVs) have crossed species to cause pneumonia. CoV causing severe acute respiratory syndrome (SARS-CoV) emerged in 2003 in Guangdong Province, China, and caused more than 700 deaths in 5 countries. The Middle East respiratory syndrome CoV (MERS-CoV) appeared in 2012 in the Arabian Peninsula, causing 858 known deaths over the last ten years, at a high case fatality rate, estimated at 35% of patients reported with MERS.^1^^,^^2^ On the other hand, the severe acute respiratory syndrome CoV-2 (SARS-CoV-2), first identified in December 2019 in the Wuhan Province, China, rapidly spread worldwide. The sharp escalation forced the World Health Organization (WHO) to declare a pandemic in March 2020. Within two years of its identification in Wuhan (as of September 2022), it had caused more than 608 million cases with 6.5 million deaths worldwide. Four others endemic CoVs of low pathogenicity affect humans: hCoV-OC43, HCoV-HKU1, HCoV-NL63, and HCoV-229E. They are seasonal viruses accounting for ∼15% of common colds.^3^

The sequence of the SARS-CoV-2 harbors 96% similarity with the CoV RaTG13 of bats and lower percentages compared to SARS-CoV (79.5%) and MERS-CoV (55%). It indicates that bats may have been the reservoir hosts for the emergence of SARS-CoV-2.^4^^,^^5^ The idea of SARS-CoV-2 as a “generalist virus” is based on positive selection acting at the base of the bat lineage that it emerged from.^6^ Direct transmission from bats to humans or the presence of an intermediate host is still a matter of discussion.^1^ SARS-CoV-2 has peculiarities from other β-Co-Vs: *(i)* a polybasic furin protease-like (RRAR/S) site between the S1 and S2 subunits of the spike glycoprotein^7^; *(ii)* the presence of novel O-glycan-binding sites flanking the protease cleavage site in the spike.^8^

The 2019 CoV disease (COVID-19) has a broad clinical and epidemiological profile.^9^ Some have mild to moderate symptoms, including fever, cough, and breathing difficulty, and many are asymptomatic. However, a relatively high percentage of infected individuals develop severe manifestations, including respiratory failure requiring mechanical ventilation and aggressive inflammatory response. Debilitating symptoms are fatigue and dyspnea seen up to two months after the viral load decreases.^10^ Individuals over 50 years old and with chronic conditions have an increased probability of evolving severely with a risk of death.

**Figure 1:**
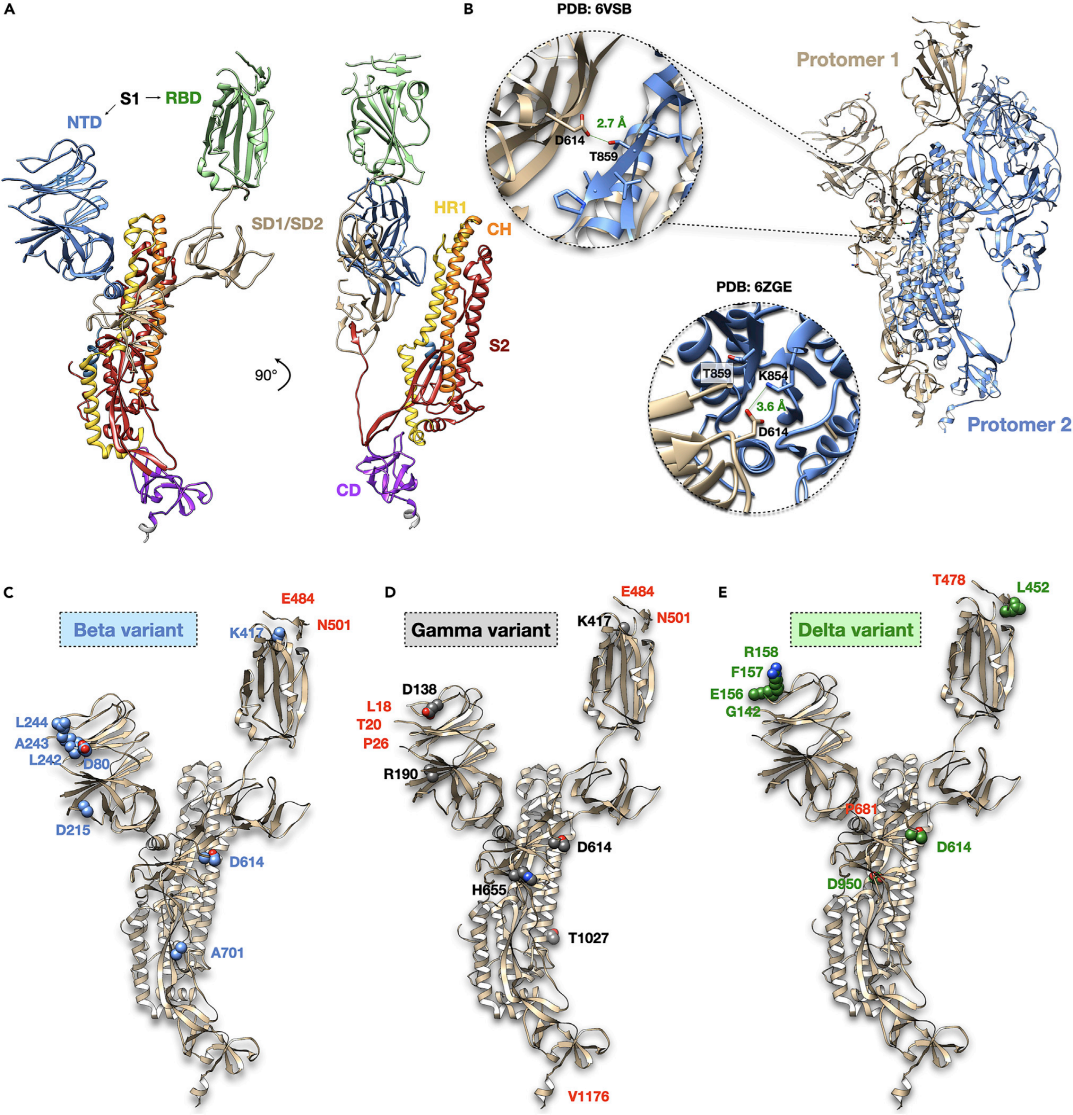
Spike glycoprotein and the variants of concern (A) Atomic model (PDB: 6VSB) showing the ribbon representation of the spike protomer obtained from cryo-EM maps. Schematics is color-coded for the N-terminal domain (NTD, blue); receptor-binding domain (RBD, green); sub-domain 1 and 2 (SD1/SD2, tan); fusion peptide (FP, turquoise); central helix (CH, orange); heptad repeat (HR1, gold yellow); S2 subunit (dark red); connector domain (CD, purple). The NTD and RBD form the S1 subunit. (B) Atomic model highlighting two spike protomers and the D614 interprotomer interaction. Zoomed images show D614-T859 H-bond interaction (PDB: 6VSB) at 2.7 Å distance and the D614-K854 salt bridge at 3.6 Å distance (PDB: 6ZGE). (C–E) Atomic model (PDB: 6VSB) showing altered sites for (C) beta variant in blue, (D) gamma variant in black, (E) delta variant in green. Altered sites are shown as spheres. Labels in red refer to residues not resolved on the cryo-EM map.

The spike glycoprotein is the leading glycoprotein at the viral envelope and the primary antigenic candidate for vaccines against COVID-19. Each protomer has two subunits, S1 (residues 14–685) and S2 (residues 686–1273), in addition to a signal peptide (residues 1–13). Essential domains of S1 are the N-terminal domain (NTD, residues 14–305) and the receptor-binding domain (RBD, residues 319–541) (Figure 1A). S2 carries the fusion peptide (FP, residues 788–806), repetitive heptapeptide sequences (HR1, residues 912–984; HR2, residues 1163–1213), the transmembrane domain (TM, residues 1213–1237), and the connector domain (CD, residues 1237–1273) (Figure 1A). The RBD binds to the host receptor, the peptidase angiotensin-2-converting enzyme (ACE 2).^7^ Cryo-EM revealed that H-bonds and salt bridges govern RBD-ACE 2 interactions.^11^ After the interaction, the S2 subunit undergoes conformational changes exposing the FP to interact with the host membrane. It leads to HR1-HR2 interactions bringing the viral envelope close to the cell’s surface,^2^ and fusion occurs when host proteases promote the cleavage of the furin-like site. Weak hydrophobic interactions drive the trimer interface, unlocked during pre/postfusion conformational positioning.^12^

Virus evolution is an issue of concern during a pandemic. Although most viral mutations are expected to be harmful, swiftly purged, or neutral, a small proportion may alter the virus biology, affecting pathogenicity, infectivity, and antigenicity.^13^ After a period of evolutionary stasis, a set of SARS-CoV-2 mutations has arisen as “variants of concern” (VOC).^6^ The initial months of the COVID-19 outbreak featured a randomly neutral and weakly purifying selection as expected at the early phases of transmission.^6^ At this stage, a naive susceptible population is not exerting any selective pressure on the pathogen.^6^ Fitness-enhancing mutations such as the D614G in the spike^14^^,^^15^ and the P323L in the RNA-dependent RNA polymerase^16^ emerged, contributing to viral transmissibility but fewer to pathogenicity.^17^ Other early detected mutations include N439K and Y453F in the spike, leading to enhanced RBM-ACE 2 affinity.^18^ N439K circulated widely in European countries (lineage B.1.258), while Y453F has been identified in Denmark over sequences related to infections in humans and minks (lineage B.1.1.298).^19^

Wide-genome search aiming to find signatures for adaptive pressure captured an increase in selective forces acting on SARS-CoV-2 genes.^20^ Adaptive pressure is a natural consequence presumably because of increasing host immunity due to infection or vaccination and the shift in the host environment due to public health intervention and social distancing. This positive selection changed the virion antigenic phenotype, and variants emerged carrying more convergent mutations that compromised the host immune recognition. In this vein, three divergent SARS-CoV-2 lineages succeeded, namely the alpha (B.1.1.7), the beta (B.1.351), and the gamma (P.1) variants.^21^^,^^22^ Known as 501Y lineages due to the persistence of an N501Y substitution in the RBD, these variants presented altered phenotypes, including increased ACE-2 affinity,^23^ transmissibility, and capacity to overcome vaccination-induced immunity.^24^ E484 is an immunodominant site with various substitutions, including the E484K present within the beta and the gamma. Together, N501Y and E484K increased RBD-ACE-2 affinity to 12.7-fold^23^ and decreased neutralization by convalescent sera,^25^ vaccine-elicited antibodies, and monoclonal antibodies.^26^^,^^27^

Mechanisms affecting antigenicity include amino acid substitutions altering epitopes and neutralizing activity, increase of the receptor-binding avidity, deletions, insertions, changes in glycosylation sites, and the presence of allosteric effects.^17^ Within the context of glycosylation changes, the T20N substitution in the gamma variant introduced a new glycosite.^28^ The lack of extensive glycosylation in RBD and its abundance in NTD has been hypothesized to play a role in RBD immunogenicity.^29^^,^^30^ In this vein, 41 monoclonal antibodies recognized the NTD and neutralized SARS-CoV-2, allowing the definition of an antigenic NTD map.^30^ The presence of several NTD substitutions, additions, and deletions indicates the domain is under selective pressure and should compose the targetable toolkit to avert SARS-CoV-2 progression.

While undergoing antigenic evolution, one question is whether authorized vaccines will maintain protection from severe disease and death for future SARS-CoV-2 variants. The temporal and geographic efficacy is an aspect requiring constant surveillance. Updated vaccines tailored to emerging antigenic variants that cross-react against several circulating variants represent an strategy to fight the pandemic. Here, we present comparative biochemical and biophysical data of the full-length ancestral and D614G spike together with three other highly transmissible strains classified by the WHO as VOC: beta, gamma, and delta. We found that during the SARS-CoV-2 adaptation, the spike glycoprotein accumulates alterations that lead to a tendency of less structural stability.

Furthermore, we revealed D614G features supporting a model of temporary fitness advantage required for virus spillover worldwide but not for long-term adaptation. The decreased trimer stability observed for the ancestral and the gamma strain and the presence of D614G uncoupled conformations is consistent to higher ACE-2 affinities when compared to the beta and delta strains. Mapping spike variants’ energetic landscape and flexibility are necessary to improve vaccine development.

## Results

### The glycosylation pattern of SARS-CoV-2 spike variants

**Figure 2:**
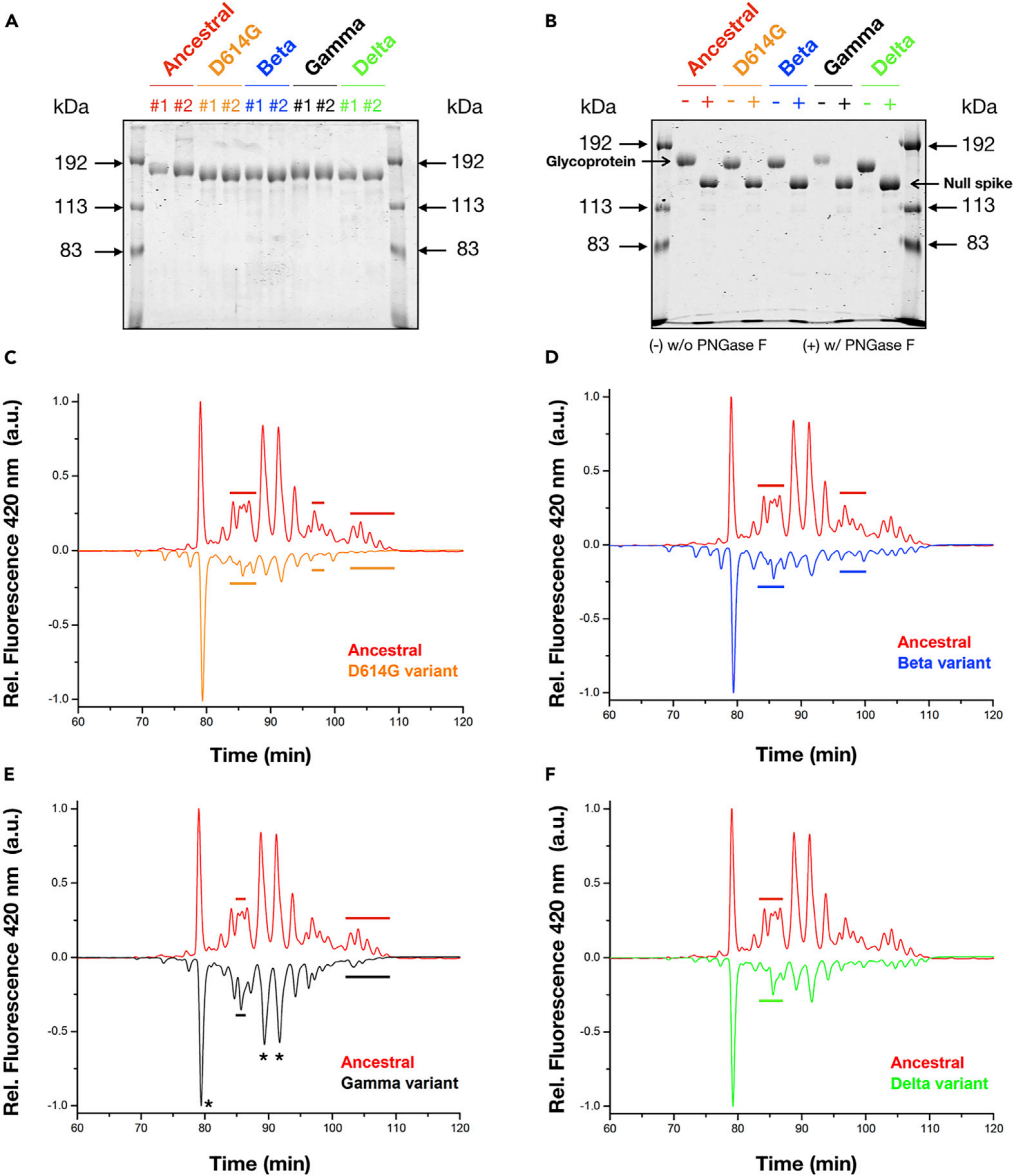
Spike protein glycosylation profile (A and B) Images of SDS-PAGE gels showing (A) two representative batches (#1 and #2) of purified spikes and (B) spikes in the absence (−) or in the presence (+) of PNGase F. Positions of the glycosylated and deglycosylated (“null”) spikes are shown in the gel. (C–F) Line plots showing the relative fluorescence as a function of time from hydrophilic chromatography (HILIC) of (C) ancestral vs. D614G, (D) ancestral vs. beta, (E) ancestral vs. gamma, and (F) ancestral vs. delta. Results are flipped to facilitate visualization. Black asterisks show abundant glycan peaks. Thick horizontal bars show major differences relative to the ancestral spike. See also Figures S1, S2, and S3.

We produced the full-length spike containing the ancestral sequence, the D614G early variant, and three highly transmissible VOCs: beta, gamma, and delta (Figures 1B–1E and Table 1). We performed quality checks to ensure our preparations’ identity and purity (Figures 2A, Figures S1, S2, and S3). A sandwich ELISA confirmed that the proteins were functional and bound to the ACE-2 receptor, and to a monoclonal antibody developed against the ancestral spike protein (Figure S4). By means of mass spectrometry analysis, we confirmed a peptide coverage higher than 94% of studied variants (Figure S1 and S2). Peak integration of size-exclusion chromatograms (SEC) showed high purity preparations (Figure S3). SDS-PAGE gels revealed a band between the reference ladders at 113–192 kDa (Figure 2A). The theoretical mass of the spike is ∼136 kDa, suggesting that the distinct mobility reflects additional mass bound to the protein.

**Table 1:** Spike variant features

Variant name	Domain	Alteration	First appearance
**Ancestral**	–	–	Nov-2019 (China)
**D614G**	SD1/SD2	D614G	Jan-2020 (China and Germany)
**Beta (B.1.351)**	NTD	D80A/D215G/del242/del243/del244	Dec-2020 (South Africa)
	RBD	K417N/E484K/N501Y	
	SD1/SD2	D614G/A701V	
**Gamma (P.1)**	NTD	L18F/T20N/P26S/D138Y/R190S	Jan-2021 (Brazil)
	RBD	K417T/E484K/N501Y	
	SD1/SD2	D614G/H655Y	
	CH	T1027I	
	After CD	V1176F	
**Delta (B.1.617.2)**	NTD	T19R/G142D/del156/del157/R158G	Apr-2021 (India)
	RBD	L452R/T478K	
	SD1/SD2	D614G/P681R	
	HR1	D950N

We noticed gentle mobility differences among purified variants. While the ancestral and gamma variants are slightly higher in the gels, the D614G, the beta, and the delta variants are lower (Figure 2A). Because the SARS-CoV-2 spike is highly glycosylated,^31^^,^^32^ we performed an enzyme-induced cleavage of N-linked glycans to show that purified spikes are in their glycosylated forms (Figure 2B).

The migration of the glycosylated ancestral and the gamma against the D614G, beta, and delta in denatured gels (Figure 2A) raise whether their glycan amount is the same. Using hydrophilic chromatography (Figures 2C–2F), we noticed that the change in the relative abundance of the three major glycan peaks is smaller between the ancestral and the gamma (Figure 2E, black asterisks) than when compared to the D614G, beta, and delta (Figures 2C, 2D, and 2F). The result is indicative that the ancestral and the gamma have a higher amount of attached glycans when compared to the others, and it goes in line with the slightly higher migration position of these proteins in denatured gels (Figure 2A). In terms of the HILIC profile, it is harder to identify pronounced differences, but some have been annotated by thick horizontal bars (Figures 2C–2F).

### Structural features of SARS-CoV-2 spike variants

**Figure 3:**
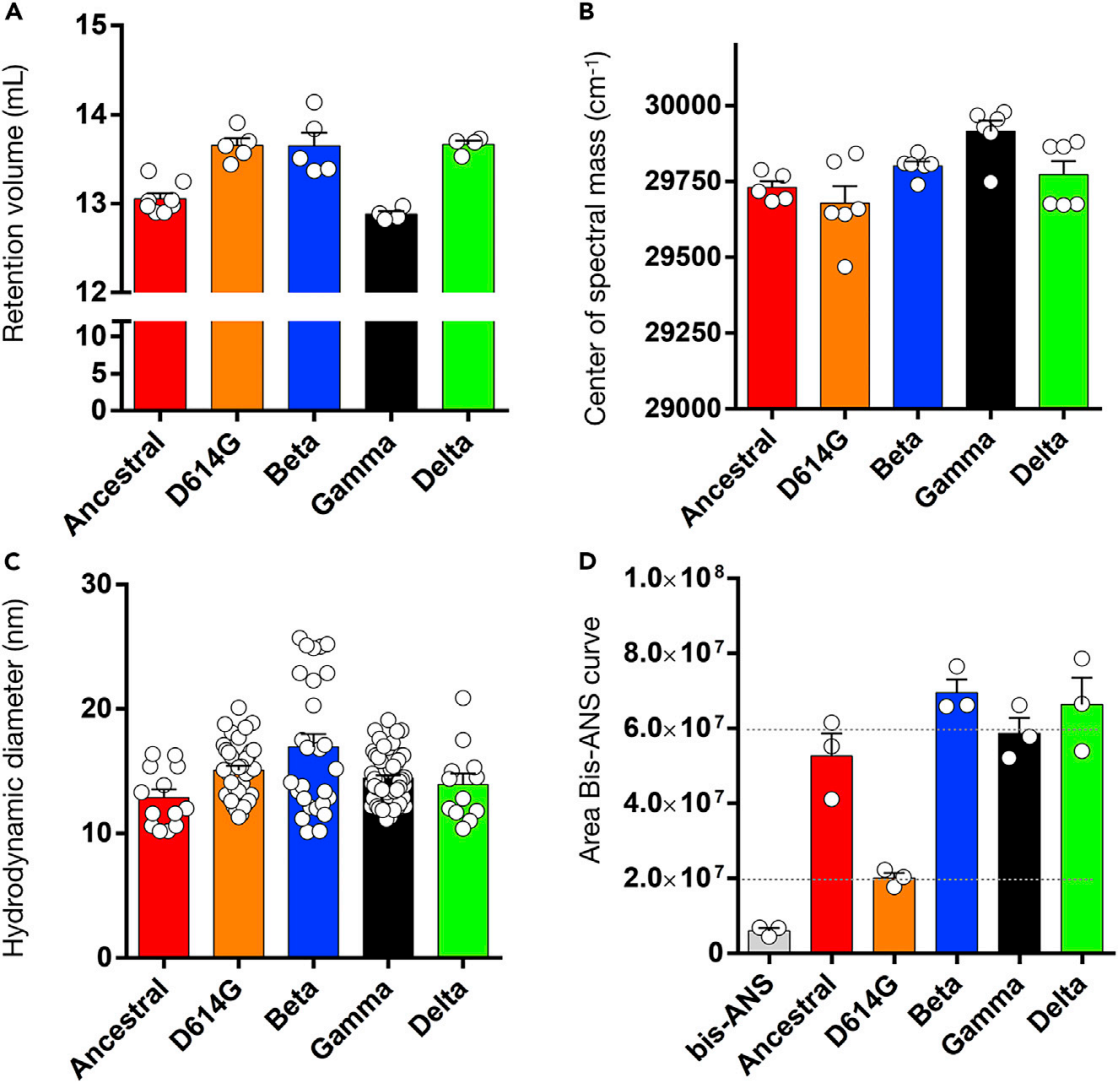
Structural features of SARS-CoV-2 spikes (A–D) Scatterplots showing (A) the retention volumes of studied spikes obtained from analytical size-exclusion chromatography. Data show the avg. ± SEM of independent protein batch preparations (n = number of data points for each series); (B) the center of spectral mass of studied spikes obtained from the emission fluorescence spectrum upon excitation at 280 nm. Data show the avg. ± SEM of at least three independent protein batches; (C) the hydrodynamic diameter of studied spikes determined by dynamic light scattering. Data are the avg. ± SD of several technical replicates from the same protein batch; (D) Area under the Bis-ANS curve of studied spikes obtained from the emission fluorescence spectrum upon excitation at 360 nm. Data show the avg. ± SEM of independent protein batches (n = 3). The gray bar shows bis-ANS fluorescence noise in the presence of buffer. See also Figures S5, S6, and S7.

We performed biophysical experiments to comparatively understand variant differences. To check whether purified proteins are forming trimers, we performed analytical SEC (Figures 3A and S5A). All five proteins revealed a retention volume close to the reference marker at 670 kDa (Figure S5A). The theoretical mass of the trimer is 408 kDa, excluding any attached glycans. Thus, the results indicate a trimeric spike in solution.

Moreover, we noticed gentle differences in the retention values among studied variants (Figure S5A). To check the robustness of those differences, we produced additional batches of each variant. We consistently observed the differences in retention values among the variants (Figure 3A and Table 2). The ancestral strain and the gamma variant showed smaller retention values when compared to the D614G, beta, and delta (Figure 3A).

**Table 2:** Biophysical parameters of spike variants

	DLS		SEC		Fluorescence			
	D_H_ (nm)	Σ > σ	Ret._vol_ (mL)	aT_50%_ (min)	*v* (cm^−1^)	G^1^_50%_ (M)	bG^2^_50%_ (M)	Bis-ANS (a.u.)
**Ancestral**	12.8 ± 0.65	5.5	13 ± 0.05	∼18	29,730 ± 20	0.049	2.24	52 ± 6
**D614G**	15 ± 0.36	5.0	13.6 ± 0.07	ND	29,678 ± 55	ND	ND	20 ± 1.3
**Beta**	16.9 ± 1	28.9	13.6 ± 0.14	ND	29,801 ± 14	0.03	1.67	69 ± 3.5
**Gamma**	14.4 ± 0.26	3.9	12.8 ± 0.03	∼43	29,915 ± 34	0.1	1.6	58 ± 4
**Delta**	13.9 ± 0.87	9.1	13.6 ± 0.04	ND	29,772 ± 43	ND	ND	66 ± 7

a Trimer-to-monomer spike dissociation at 40°C. (n.d. not detected).

b Refers to monomer stability.

To explore whether surface differences and the altered polypeptide sequence would impact the structure of the variants, we evaluated the secondary and tertiary structure content by circular dichroism (Figure S5B) and intrinsic fluorescence spectroscopy (Figures 3B and S6A, S6C, S6E, S6G, and S6I), respectively. Using dynamic light scattering (DLS), we measured the overall hydrodynamic spikes’ diameter (Figures 3C and S7A–S7E). Finally, we used fluorescence to measure the surface exposure of the proteins’ hydrophobic motifs (Figures 3D and S6B, S6D, S6F, S6H, and S6J). Far-UV circular dichroism spectra showed the expected behavior for an alpha-helix enriched protein with negative peaks at 222 and 208 nm and no differences among studied variants (Figure S5B). Fluorescence emission spectra upon excitation at 280 nm revealed λ_max_ of ca. 320 nm for all variants, but gentle differences in fluorescence intensity at λ_max_ (Figures S6A, S6C, S6E, S6G, and S6I). The center of spectral mass (*ν*) is sensitive to inform whether excited probes (mostly Trp residues) are more exposed or hidden into the spikes’ structure. The smaller the value, the more exposed the Trp residues are to the surface. No significant differences in *ν* values have been captured, indicating that the overall spikes’ structure is folded (Figure 3B and Table 2). Hydrodynamic diameter (D_H_) measurements revealed avg. ± SEM values of 12.8 ± 0.65 nm (n = 13), 15 ± 0.36 nm (n = 39), 16.9 ± 1 nm (n = 27), 14.4 ± 0.26 nm (n = 55), and 13.9 ± 0.87 nm (n = 12) for the ancestral, D614G, beta, gamma, and delta, respectively (Figure 3C and Table 2). The beta strain showed the highest D_H_ variance (σ) value (σ = 28.9). The second highest value was for the delta strain (σ = 9.1), suggesting that these proteins show more heterogeneous structures in solution (Figure 3C and Table 2).

To learn about the content of hydrophobic motifs on the proteins’ surface, we measured the emission fluorescence spectra of a molecular probe (bis-ANS). Bis-ANS significantly increases its quantum yield upon binding to exposed hydrophobic protein surfaces (Figures 3D and S6B, S6D, S6F, S6H, and S6J) that may reflect traces of non-native spike monomers or oligomeric species. When bis-ANS is free in solution, the spectrum gives a λ_max_ of ca. 530–540 nm but undergoes a blue-shift to 470–490 nm and an intensity increment upon binding (Figure S6K). In contrast to all other spikes, the D614G presented a 3-fold bis-ANS intensity reduction suggesting that the Asp-to-Gly substitution at position 614 can reduce the hydrophobic motifs’ surface exposure (Figure 3D and Table 2). It is worth mentioning that, except for the ancestral spike, all other studied variants have the D614G substitution, which made us conclude that the additional alterations of the beta, gamma, and delta could revert the lower hydrophobic surface exposure of the D614G variant. We did not detect traces of monomers or oligomeric species by SEC, suggesting that reported bis-ANS fluorescence is more likely due to differences in exposed hydrophobic patches at the surface of trimeric spike.

### Modulating the structural stability of SARS-CoV-2 spike and variants

**Figure 4:**
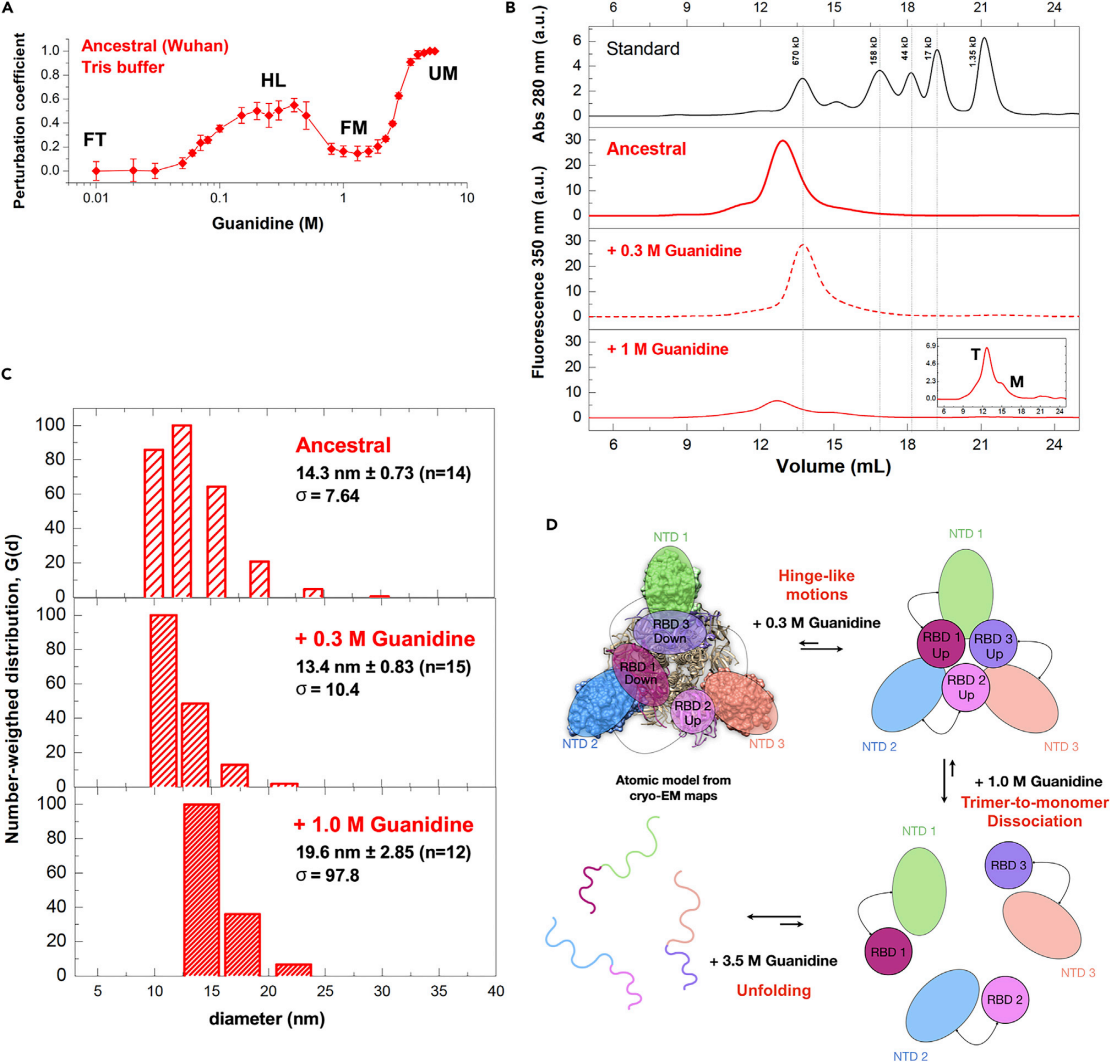
Structural stability of the ancestral spike (A) Dot plots showing the perturbation coefficient as a function of guanidine increments for the ancestral spike in the presence of tris-based buffer (tagged protein, see STAR methods for details). Abbreviations on panel A stand for FT, folded trimer; HL, hinge-like; FM, folded monomer; UM, unfolded monomer. Data are represented as avg. ± SEM of (n = 3, independent protein batches). Check Figure S8Bb. (B) Line plots showing SEC runs for the ancestral spike in the presence of the tris-based buffering (thick red line) and in the presence of 0.3 M (dashed red line) and 1 M of guanidine (thin red line). Insert shows the y-scaled chromatogram at 1 M of guanidine to visualize the peaks corresponding to the trimer (T) and the monomer (M). Standard is colored black. (C) DLS number-weighted distribution as a function of hydrodynamic diameter in the absence (ancestral) and presence of 0.3 and 1 M of guanidine. (D) Schematics showing spike top views to illustrate the interpretation of the data. At 0.3 M of guanidine, the data supports RBD hinge-like motions. At this stage of knowledge, we cannot rule out whether all three RBDs are facing up. At 1 M of guanidine, the data indicate trimer-to-monomer dissociation. At concentrations higher than 3.5 M guanidine, monomer unfolding is achieved. See also Figure S8.

**Figure 5:**
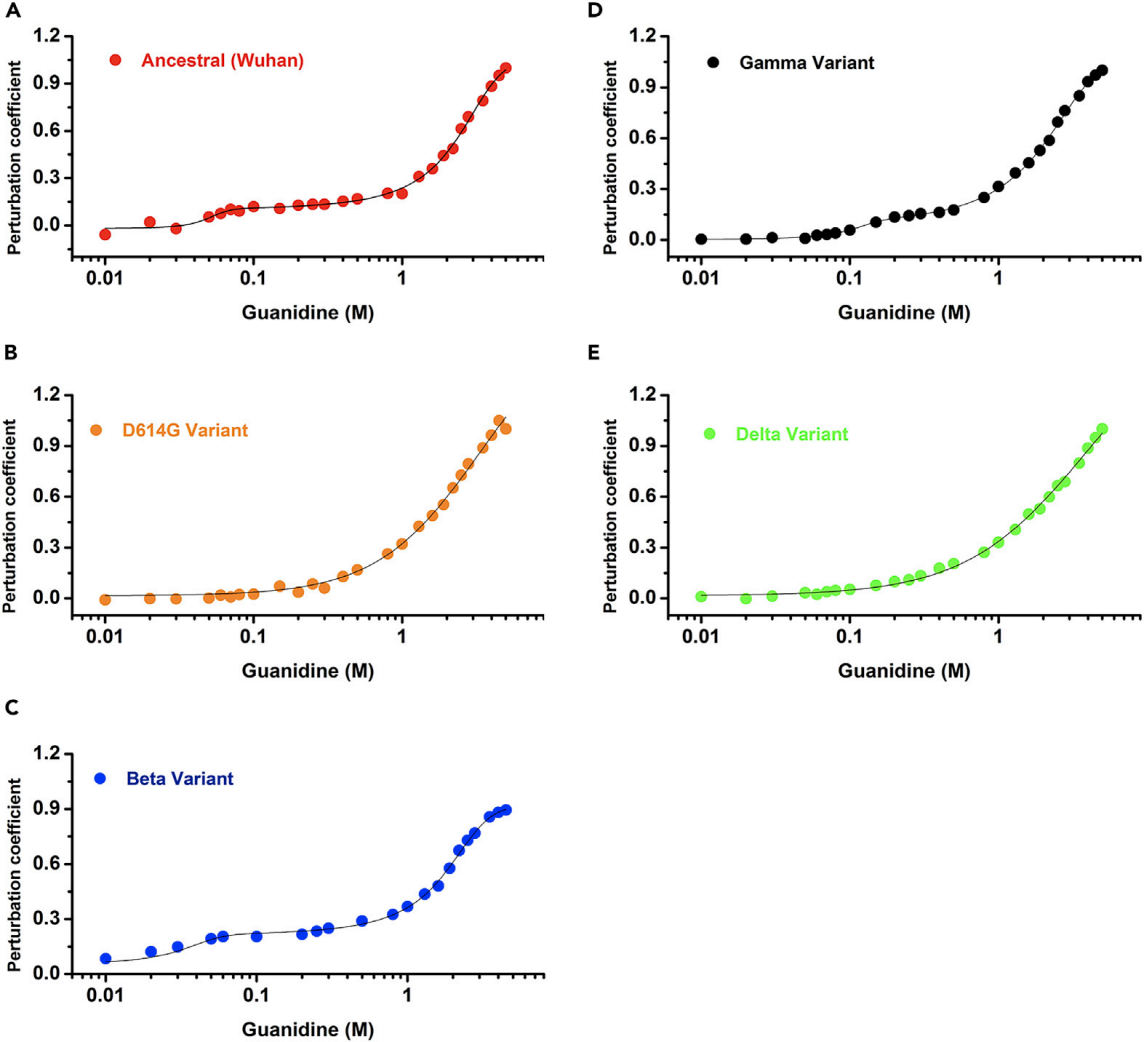
Structural stability of spike variants Dot plots showing the perturbation coefficient as a function of guanidine increments for the (A) ancestral, (B) D614G, (C) beta, (D) gamma, and (E) delta variants. Curves were carried out in phosphate buffer and tagless proteins (see STAR methods for details).

Because structural stability of surface antigens may have a role in transmissibility tendencies,^33^^,^^34^ we decided to explore the stability of spike variants by using chemical and physical strategies. We first attempted to find buffer compositions to discriminate energetic gaps related to conformational changes, dissociation, and unfolding events when challenging the ancestral spike with increasing concentrations of guanidine. We noticed that a tris-based buffer vs. phosphate buffering leads to drastic changes in chemical-induced titrations (Figures 4 and 5). While phosphate buffer showed two- and three-state transitions for studied variants (Figures 5A–5E), a tris-based buffer (see STAR methods) revealed an unexpected four-state transition for the ancestral spike (Figure 4A and Figures S8A-Aa). The data of spike variants in Tris buffer were not included in our analysis due to sample limitations. To check the consistency of the four-state transition, we produced additional ancestral batches, and all reproduced the four-state profile (Figure S8Bb). The reliability of the ancestral traces motivated us to explore the enriched species in each transition. In the first-transition range, the fluorescence emission spectra showed a gentle intensity increase followed by a smooth red-shift (Figures S8A–S8M, from 0 up to 0.3 M). The next transition was a delicate blue-shift accompanied by an intensity reduction (Figures S8N–S8T, from 0.4 to 1.9 M). Finally, we noticed a strong red-shift from λ_max_ at ca. 330–356 nm, possibly explained by an unfolding process in which the intrinsic probes moved away from hydrophobic moieties into the solvent (Figures S8U–S8Aa).

Using the ancestral strain, we performed analytical SEC and DLS at 0.3 and 1 M of guanidine to learn about the second and third plateau stages (Figures 4B and 4C). At 0.3 M of guanidine, the trimeric spike showed a larger retention volume when compared to the folded trimer (Figure 4B) and, in agreement with that, an amenable decrease in average D_H_ values (Figure 4C). The SEC and DLS data suggest a tight trimer at 0.3 M of guanidine that would indicate hinge-like motions occurring in the spikes’ structure. At 1 M of guanidine, SEC and DLS revealed a minor peak consistent with a trimer-to-monomer dissociation (Figure 4B) and more considerable variance and D_H_ values pointing to the formation of more heterogeneous species in solution (Figure 4C). Additionally, chromatograms presented a full width at half maximum of 1.63, 1.55, and 2.98 for the spike in the absence and presence of 0.3 and 1 M of guanidine, respectively (Figure 4B). These values suggest spike displaces from a narrow ensemble of species at 0.3 M to a broader ensemble at 1 M, consistent with an equilibrium shift to trimer dissociation. Figure 4D summarizes the proposed events occurring during the chemical-induced reaction recorded by fluorescence spectroscopy.

Hereafter we focused on the folding-unfolding equilibrium to understand the stability of the variants. We performed chemical-induced reactions with phosphate in which the major detected species are folded trimers and unfolded monomers. The curves revealed two distinct profiles. While the ancestral, beta, and gamma variants exhibited a biphasic shape, D614G and delta undergo a single transition (Figures 5A–5E). The G^2^_50%_ values in which half of the spike monomers are unfolded revealed the order of stabilities: ancestral > beta > gamma (Table 2). The D614G and delta curves did not reach a plateau stage at 5M guanidine (Figures 5B and 5E), indicating they are more stable than the others.

### Modulating SARS-CoV-2 spike by physical forces

**Figure 6:**
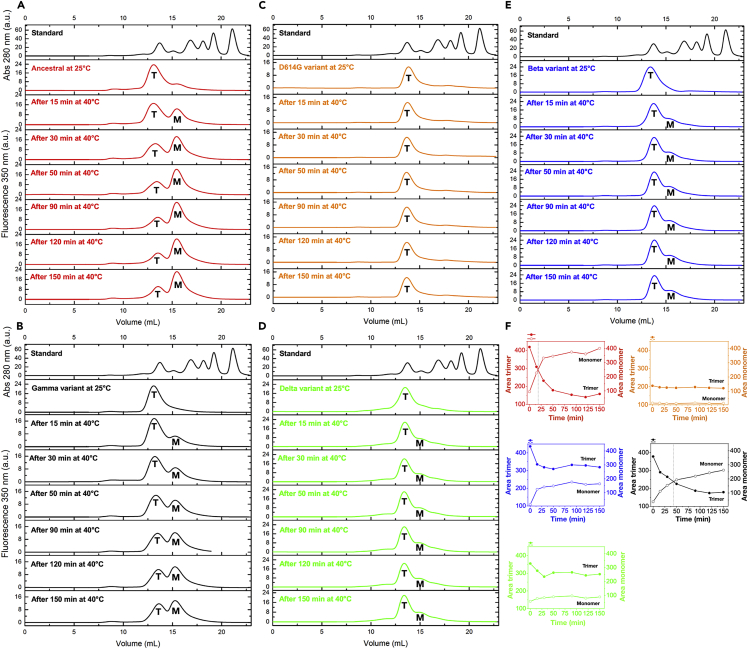
Trimer-to-monomer spike dissociation (A–E) Collection of line plots showing the fluorescence signal at 350 nm as a function of retention volume for (A) the ancestral strain in red, (B) gamma in black, (C) D614G in orange, (D) delta in green, and (E) beta in blue at 25°C and after several times at 40°C. T and M stand for trimer and monomer, respectively. The absorbance at 280 nm of the calibration standard is shown at the top of each experiment. (F) Double-Y plots show the integration area corresponding to the trimers (filled dots) and the monomers (empty dots). The color code is the same as for panels (A–E). Crossover points (T_50%_) are highlighted by vertical dashed lines for the ancestral strain (red) and gamma variant (black).

We designed experiments to understand the trimer-to-monomer equilibrium of each variant. We captured their dissociation tendencies by increasing the incubation time to 40°C, followed by SEC injections at room temperature (Figures 6A–6E). For the ancestral and the gamma, the peak corresponding to the trimeric spike (T) decreased concomitantly to the increase of the spikes’ monomeric peak (M) (Figures 6A and 6B). The D614G has not shown any traces of trimer dissociation within the evaluated time frame (Figure 6C). In contrast, the beta and delta variants revealed smaller amounts of monomers resulting in a shoulder-like peak (Figures 6D and 6E). The peak areas of the trimers and monomers as a function of time showed crossover points for the ancestral and the gamma variants (Figure 6F, red and black plots). The crossover point indicates the T_50%_ when the reaction has approximately 50% of trimers and monomers in the solution. The T_50%_ values were ca. 18 and 43 min for the ancestral and the gamma, respectively.

We also evaluated the effects of hydrostatic pressure as a physical force acting primarily on the volumetric properties of macromolecules.^35^ By following the bis-ANS fluorescence as a function of pressure, we noticed that the delta variant is more susceptible to pressure-induced hydrophobic exposure when compared to the others (Figures S9A–S9F). All spike versions showed a similar tendency to decrease approximately 30% of the scattering intensity against pressure increments, except for the beta variant, which showed a lower decrease as a function of the pressure exerted (Figure S9G–S9L). The results are consistent with a trimer dissociation, with the delta showing the highest susceptibility of hydrophobic exposure during dissociation (Figure S9F) and the beta showing the lesser dissociation tendency (Figure S9L).

## Discussion

The data revealed features between the gamma and the ancestral spike. They present equal mobility shifts on denatured SDS-PAGE gels likely because of their similar glycan abundance (Figure 2). Moreover, they share similar retention volumes (Figure 3A) and a detectable pattern of trimer-to-monomer dissociation despite different T_50%_ values (Figures 6A and 6B). These features are unique to the ancestral and gamma spikes and not observed for the D614G, beta, and delta variants. Comparatively, the ancestral, the D614G, and the gamma revealed a higher affinity to ACE-2 than the beta and the delta (Figure S4). The D614G variant showed unique features such as a 3-fold reduction in bis-ANS fluorescence (Figure 3D) and no trimer dissociation after 150 min at 40°C (Figure 6C). Furthermore, the beta variant showed the highest variance in D_H_ values, followed by the delta (Figure 3C).

Using chemical-induced unfolding, we provide evidence to argue that during SARS-CoV-2 adaptation, the spike monomer accumulates changes that lead to less structural stability for some variants such as the beta and gamma (Figure 5). *In silico* data have shown that SARS-CoV-2 was selected for amino acid substitutions, resulting in more stable spikes.^36^ Still, a significant drawback of this study is the absence of glycosylation effects on protein stability.^36^ Our results point opposite, and data are provided using glycosylated spikes. We also captured energetic gaps for the ancestral spike that is reasonably due to hinge-like motions, trimer dissociation, and unfolding (Figure 4). The sequential events of conformational change and trimer dissociation in unfolding ancestral spike open insights into understanding more general protein folding mechanisms in complex biological systems. However, we cannot distinguish which spike domain undergoes conformational changes prior to trimer dissociation.

The features of D614G are intriguing. D614G-containing viruses replaced the ancestral strain to reach worldwide near-fixation within a few months after the COVID-19 pandemic started.^14^ This substitution became dominant by April 2020^14^ and appeared to be a fitness advantage rather than genetic drift as its frequency increased globally and during co-circulation within individual regions.^15^ SARS-CoV-2 carrying G614 enhances viral replication in human lung cells, expanding the infectivity and stability of virions.^15^ Furthermore, G614-infected hamsters produced higher infectious titers in nasal washes and the trachea, supporting higher viral loads in the upper respiratory tract of patients with COVID-19.^15^^,^^37^ Structures from April 2020 revealed that the D614 of one protomer forms an H-bond with the T859 at the S2 of the adjacent protomer.^7^^,^^38^ However, structures from August 2020 revealed that D614 contacts K854, forming a salt bridge^39^ (Figure 1B). The shorter G614 side chain leads to a dilated packing and a range of open conformations, presumably because of increased flexibility and local unfolding. Cryo-EM studies on D614 (ancestral) and G614 spikes revealed a flip in the ratio of close-to-opened conformations.^40^ The D614G structural flexibility seems to be related to its higher infectivity, and G614 persists in all subsequent variants that emerged. However, we observed features of D614G that are not shared among the studied VOCs. Hence, the D614G mutation was a relevant aspect of the virus spreading at the early stages of the pandemic. Compared with the ancestral, beta, gamma, and delta strains, the D614G variant was the only one to present a substantial reduction in surface exposure of hydrophobic motifs (3-fold) and trimer persistence after 2 h under mid-temperature incubation (40°C). Less hydrophobic surface exposure means that water is weakly repelled, which may help create a relaxed topology for heterogeneous ensembles and multiple states of openness.

Several hypotheses attempted to explain the effects of D614G for infectivity and transmissibility. One is that the protomer impairment would cause local unfolding and prime the fusion peptide to a fusion-prone conformation.^41^^,^^42^ Another possibility is that local unfolding may trigger the exposure of asparagine sites amenable to the glycosylation machinery.^43^ Glycan modification has been shown to modulate spike conformation, stability, and host cell invasion.^44^^,^^45^^,^^46^ Recently, N-glycan at position N343 revealed a gating role facilitating RBD opening,^45^ and surprisingly, D614G has shown substantial differences in glycan content at N343 compared with the WA1 ancestral strain.^43^ Other study comparing the N-glycosylation spike profile of the ancestral and D614G under identical conditions revealed that D614G decreased the relative abundance of complex-type glycans and increased the oligomannose glycans leading to an overall reduction of the glycosylation complexity.^47^ This finding aligns with our HILIC profile showing differences between the ancestral and D614G variant (Figure 2C).

The less hydrophobic surface exposure (Figure 3D) and trimer persistence (Figure 6C) with D614G populating more open and flexible conformations^40^^,^^41^ support the screening flexibility vs. higher immunogenicity models for fitness advantage. It is worth mentioning that D614G reduced ACE2 affinity due to a faster dissociation rate,^40^ supporting the screening flexibility model. Weaker antigen stiffness generates a more structurally adaptable antigen with potential unlocked domain conformations. This phenomenon would help on two sides: screening more rapidly for potential ACE-2 interacting surfaces and binding more tightly once required. This interpretation reconciles apparent controversial results on D614G affinity for ACE-2. In our hands, the ancestral, the D614G, and the gamma revealed higher ACE-2 affinity when compared to the beta and delta spikes as measured by EC_50_ values (Figure S4). The beta and delta variants have a 2-fold affinity increase to ACE-2 when tested against isolated RBD domains.^48^^,^^49^ We used the full-length trimeric and glycosylated spikes, which might likely explain the apparent contradiction. A recent study revealed that the glycosylation content also impacts receptor binding,^50^ and our HILIC data show different glycan content for the ancestral and gamma compared to the others. Thus, glycosylation would play a role for the observed difference in EC_50%_ values.

D614G is probably a trade-off between a structurally adaptable antigen promoting a more significant advantage for ACE-2 screening at the cost of higher immunogenicity. Although D614G substitution persisted in subsequent variants, the beta, gamma, and delta reversed the lesser hydrophobic exposure (Figure 3D), presumably due to the acquisition of other alterations (Figures 1C–1E). Other works explored the thermostability of D614G, which aligns with the SEC data at mid temperatures (Figure 6C). The increased D614G trimer thermostability is presumably explained by an entropic compensation that is revealed by higher conformational heterogeneity contributing to the free energy gain of the system.^51^^,^^52^ Altogether, we conclude that the decreased hydrophobic exposure and higher flexibility of D614G were critical for the virion to achieve fast worldwide near-fixation but irrelevant for long-term virus adaptation.

The ancestral and the gamma spikes share similar retention volumes (Figure 3A) and equal mobility shifts on denatured SDS-PAGE (Figure 2A) primarily due to corresponding glycan abundance (Figure 2E). Alternatively, they would ensemble distinct conformational states when compared to the others, but the HILIC data support the glycan contribution (Figures 2C–2F). They also have a discernible trimer-to-monomer dissociation pattern despite different T_50%_ values (Figures 6A and 6B). These shared properties are significant because they are not observed for D614G, beta, and delta variants. Recent biochemical results revealed that the gamma protein eluted in three distinct peaks, corresponding to the prefusion trimer, postfusion trimer, and dissociated monomers, with the prefusion trimer corresponding to less than 40% of the total protein, similarly to the ancestral protein.^28^^,^^53^ The authors concluded that the trimer is not stable for this variant and raised uncertainty of why the gamma trimer dissociates.^28^ In our hands, the gamma and the ancestral trimer are eluted as a single peak, probably related to the mutations used in our gene construct to stabilize the protein trimer in the prefusion conformation.^38^ However, we detected a trimer-to-monomer dissociation profile for both spikes (and not for the other studied variants), which is in line with the reported trimer instability for the ancestral strain and the gamma variant.^28^^,^^53^

Notwithstanding, the gamma trimer is more stable than the ancestral trimers, with T_50%_ values of 43 and 18 min, respectively. We believe the corresponding abundance of glycans bound to the surface of the ancestral and the gamma may exert, to some extent, a role in their trimer dissociation profile. It is worth mentioning that despite providing structural shielding, glycans are highly hydrophilic molecules, attracting water to their boundaries, which may contribute entropically to nucleate local dissociation at mid temperatures. Alternatively, the presence of H655Y at the S2 would contribute to destabilizing the gamma trimer.^28^ We recognize that different expression systems will generate spikes containing distinct glycosylation profiles. This situation has been shown for HEK293 vs. VERO E6 cells^43^ or HEK293 vs. baculovirus-insect Hi5 cells.^54^ Nevertheless, we also understand that different spike sequences produced in the same host cell may lead to other glycosylation profiles. The sequence-dependent glycosylation has been shown for the ancestral vs. D614G spikes.^47^ Furthermore, small changes within the 611-LYQD-614 motif also impact the glycosylation pattern of the spike protein.^55^ Our HILIC data point that sequence changes occurring during spike evolution also affect the spike glycosylation profile.

The distinction of each step on chemical-induced unfolding is relevant to understanding more general mechanisms of protein unfolding and association in complex systems. The data support a sequential unfolding model in which trimer dissociation is preceded by conformational changes to unlock quaternary spike interactions. Following this, protomer unfolding happens cooperatively. It is worth mentioning that experiments are under equilibrium and represent steady-state measures at each guanidine concentration. Thus, plateau states are likely explained by a population-weighted average of multiple conformers enriched and trapped in solution and not by a single conformer.

In summary, there is an utmost necessity to explore the dynamics of the spike variants and the way conformational changes, glycosylation, and trimer stability would affect the ensemble of spike conformations and the status of up/down RBD positioning. For example, the Folding@home platform has contributed tremendously with 0.1 s of simulation data.^56^ This study predicts spike has a trade-off between making ACE-2 binding interfaces accessible and masking epitopes to circumvent immune response.^56^ Another study used single-molecule fluorescence resonance energy transfer (smFRET) to observe spike dynamics on virus particles. The authors found that spike samples four distinct conformational states forming an intermediate during ACE-2 recognition.^57^ Finally, hydrogen-deuterium exchange coupled with mass spectrometry revealed a spike conformation that interconverts slowly with the prefusion state and is more likely explained by an open trimer that exposes the S2 trimer interface.^12^ We experimentally compared the ancestral, D614G, beta, gamma, and delta variants to uncover their trimer stability and monomer structural stability and understand spike evolution during the pandemic. Studies on the energetic landscape of the full-length spike variants in its glycosylated form are sparse but *sine qua non* to understand the antigenicity of circulating strains and help develop more effective vaccines.

### Limitations of the study

Caveats of this work include more profound evidence to understand what are the molecular determinants that lead to different glycosylation amounts of each variant and their glycosylation content.

## STAR★Methods

### Key resources table

**Table:** 

REAGENT or RESOURCE	SOURCE	IDENTIFIER
**Antibodies**		

ACE-2 Fc protein	GenScript	Cat# Z03484-1, Lot# B2011010
anti-human IgG (Fc specific)-peroxidase antibody	Sigma-Aldrich	Cat# SAB3701282-2 MG, Lot# RI34900

**Biological samples**		

HEK293 3F6	Canada	N/A

**Chemicals, peptides, and recombinant proteins**		

SARS-CoV-2 Wuhan Spike protein	Alvim et al., 2020^58^	10.1101/2020.07.13.20152884
SARS-CoV-2 D614G Spike protein	This paper	N/A
SARS-CoV-2 Gamma Spike protein	This paper	N/A
SARS-CoV-2 Beta Spike protein	This paper	N/A
SARS-CoV-2 Delta Spike protein	This paper	N/A
HEK-TF medium	Xell, Germany	N/A
HRV-3C protease	Thermofisher	#88946
SDS	USB	#75819
Triethylammonium bicarbonate	Sigma	#T7408
Dithiothreitol	Bio-Rad	#161-0611
Iodoacetamide	Bio-Rad	#163-2109
Phosphoric acid	Millipore, EMD	#1.00264.1000
Methanol	Tedia	#MS1922-001
Trypsin	Promega	#V511A
Gel filtration standard	Bio-Rad	#151-1901
Blue dextran	Sigma	#D5751
BSA	Sigma	Cat# A9647-50G, Lot# SLCH5268
TMB substrate	Life Technologies,	Cat# 2023, Lot# 11323202-7
4,4′-dianilino-1,1′-binaphthyl-5,5′-disulfonic acid, bis-ANS	Invitrogen	#B153
Guanidine	Merck	#607-148-00-0

**Critical commercial assays**		

PNGase-F	New England BioLabs	NEB #P0704S
QuBit Assay	Thermo Scientific	#Q33212
Pierce™ BCA Protein Assay Kit	Thermo Scientific	Cat# 23227, Batch# VH311372

**Recombinant DNA**		

Wuhan ancestral gene construct	GenBank	MN908947
D614G gene construct	GenScript, USA	N/A
Beta gene construct	GenScript, USA	N/A
Gamma gene construct	GenScript, USA	N/A
Delta gene construct	GenScript, USA	N/A

**Software and algorithms**		

OriginPro 8.1	OriginLab	https://www.originlab.com/
GraphPad Prism version 8	GraphPad Software	San Diego, California, USA
Proteome Discoverer 2.4	Thermofisher Scientific	N/A

**Other**		

pαH vector	B. Graham, VRC/NIH; BEI	#NR-52563
pCIneo vector	Promega	USA
S-Trap columns	Protifi	#C02-mini-80
StrepTrap XT column	Cytiva, Sweden	N/A
Acclaim PepMap	Thermo Scientific	#164946
EASY-Spray™ Column PepMap	Thermo Scientific	# 03-251-871
Superose 6 increase 10/300 GL	Cytiva	#29091596

### Resource availability

#### Lead contact

Further information and requests for resources and reagents should be directed to and will be fulfilled by the lead contact, Guilherme A. P. de Oliveira, PhD (gaugusto@bioqmed.ufrj.br).

Institute of Medical Biochemistry Leopoldo de Meis, National Institute of Science and Technology for Structural Biology and Bioimaging, National Center of Nuclear Magnetic Resonance Jiri Jonas, Federal University of Rio de Janeiro, Rio de Janeiro, RJ 21941-902, Brazil.

#### Materials availability

All requests for resources and reagents should be directed to and will be fulfilled by the lead contact, Guilherme A. P. de Oliveira, PhD (gaugusto@bioqmed.ufrj.br).

### Experimental model and subject details

Stable, recombinant HEK293 cell lines were generated to produce each spike protein by transfecting the suspension-adapted HEK293-3F6 (NRC, Canada) parental cell line with each plasmid described. After the selection of stable transfectants, the resulting recombinant cell pools were cultured in HEK-TF medium (Xell, Germany) in a CO_2_ incubator at 37°C and 5% CO_2_ using shake flasks under orbital agitation (140 rpm, 2.5-cm stroke) or in 1.5-L stirred-tank bioreactors at setpoints of pH, temperature and dissolved oxygen of 7.1, 37°C and 40% of air saturation, respectively (EzControl, Applikon, The Netherlands). Cell concentration and viability were determined by trypan blue exclusion using an automated cell counter (Vi-Cell, Beckman Coulter), whereas glucose and lactate concentrations were monitored using a metabolite analyzer (YSI 2700, Yellow Springs Instruments). Spot blots determined the presence of spike protein in the supernatants as described earlier.^58^

### Method details

#### SARS-CoV-2 spike protein constructs

The Wuhan ancestral gene construct (GenBank MN908947) encodes the ectodomain (amino acids 1-1208) spike protein in the trimeric form and the prefusion conformation.^38^^,^^58^ A pαH vector comprising this gene was kindly provided by B. Graham, VRC/NIH, and is also available from BEI Resources under #NR-52563. The sequence contains proline substitutions at residues 986 and 987, a GSAS substitution at the furin cleavage site (residues 682-685), a C-terminal T4 fibritin trimerization motif, an HRV-3C protease cleavage site, a TwinStrepTag, and an 8xHis tag. Since the pαH vector does not have a cassette for selecting stable transfectants, it must be co-transfected with an empty vector containing the neomycin phosphotransferase selectable marker.^38^

The genes coding for the D614G, beta, gamma, and delta variants were synthesized at Genscript (Piscataway, USA) and cloned into the pCIneo vector (Promega, USA) to facilitate stable expression in mammalian cells. The variants contained the respective mutations as follows:

• D614G: Asp-to-Gly substitution at position 614 (D614G);
• Beta (lineage 20H or B.1.351): D80A, D215G, 242-244del, K417N, E484K, N501Y, D614G, A701V;
• Gamma (lineage 20J or P.1): L18F, T20N, P26S, D138Y, R190S, K417T, E484K, N501Y, D614G, H655Y, T1027I, V1176F;
• Delta (lineage 21A or B.1.617.2): T19R, G142D, 156-157del, R158G, L452R, T478K, D614G, P681R, D950N;

#### Production and purification of spike variants

Cell-free HEK293 supernatant was obtained by microfiltration with 0.45-μm PVDF membranes and injected into a 5-mL StrepTrap XT affinity chromatography column (Cytiva, Sweden) following the manufacturers’ instructions using an Äkta Purifier system (Cytiva, Sweden). The purification tag was removed using HRV-3C protease (Thermofisher, #88946) to yield the tagless protein unless otherwise stated. Protein concentration, purity, and identity in the eluted fractions were confirmed by NanoDrop (Thermofisher USA), SDS-PAGE, and Western blot analyses, respectively. An extinction coefficient of 135,845 M^−1^ cm^−1^ and a molecular mass of 136,908.9 Da (monomer of the polypeptide chain) was used for the Nanodrop setting. The purified protein obtained in the affinity chromatography was stored at −80°C and thawed when needed for experiments.

#### SDS-PAGE of protein samples

We evaluated the purified spikes by 7% SDS-PAGE gels stained with Coomassie brilliant blue R250. We digitized the gels using the Odyssey scanner and software (LI-COR Biosciences). In selected samples indicated in Figure 2B, we carried out PNGase F digestion following the manufacturers’ instructions (New England BioLabs, NEB #P0704S). Control samples followed the same procedure except for the enzyme.

#### Hydrophilic interaction liquid chromatography (HILIC-HPLC)

First, we digested spike samples with PNGase F using a commercial kit (New England BioLabs, NEB #P0704S). We adopted a modified protocol as follows: a sample volume of 40 μL at 0.5 mg/mL (20 μg of spike protein) was mixed with 10 μL of denaturing buffer (part of the kit, “10X”) and incubated for 10 min at 100°C. After cooling down, 10 μL of NP-40 solution, 10 μL of GlycoBuffer, and 1 μL of the PNGase F enzyme (all kit components) were added and incubated at 37°C for 2 h. After that, we separated the free glycans by precipitating the remaining proteins with 300 μL of cold ethanol for 15 min at −20°C. The tubes were centrifuged, and the supernatants of each sample were collected. The precipitation step was repeated twice, and a pool of three supernatants collected per sample was dried in a vacuum concentrator (Eppendorf, Germany). To label glycans with a fluorophore, each dried sample was mixed with 5 μL of a solution containing 0.048 g/mL of 2-aminobenzamide (2-AB) and incubated at 65°C for 2 h. 2-AB-labeled glycans and unbound 2-AB were separated by filter paper chromatography (Whatman) using acetonitrile. 2-AB-glycans were recovered from cropped filter papers after washing them with 500 μL of ultrapure water for 10 min. We performed the washing procedure four times to produce a final volume of 2 mL (4 x 500 μL). Samples were filtered with 0.45-μm PVDF membranes and dried overnight using a vacuum concentrator (Eppendorf, Germany).

Dried samples were resuspended with 40 μL of ultrapure water and 160 μL of acetonitrile. Half of this volume was directly injected at 0.4 mL/min into an HPLC system (Shimadzu, Japan) fitted with a TSK gel amide 4.6 mm × 25 cm, 5 μm column (Tosoh Bioscience) previously equilibrated with 20% of 50 mM ammonium formate pH 4.4 and 80% of acetonitrile HPLC grade (initial step). Glycans were recorded by fluorescence at 420 nm upon excitation at 330 nm using an RF-10A XL fluorescence detector from Shimadzu. Elution was accomplished using a linear gradient in which the initial step achieved 53% of 50 mM ammonium formate pH 4.4 and 47% of acetonitrile after 132 min.

#### Mass spectrometry sample preparation

Each spike variant (25 μg) was mixed with an equivalent volume of 10% SDS(SDS, USB #75819) and 100 mM triethylammonium bicarbonate buffer, pH 8.5 (TEAB, Sigma #T7408). Disulfide bonds were reduced by incubating the samples at 90°C for 10 min with dithiothreitol at a final concentration of 10 mM (DTT, Bio-Rad #161-0611), followed by incubation at 37°C for 1 h. Samples were then incubated with iodoacetamide at a final concentration of 40 mM (IAA, Bio-Rad #163-2109) at room temperature in the dark for 30 min to alkylate disulfide bonds.

Phosphoric acid at 12% (Millipore, EMD #1.00264.1000) was added until each sample’s final concentration of 1.2%. Then, 350 μL of binding/washing buffer (100 mM TEAB in 90% methanol, Tedia #MS1922-001) was added, and the total volume was loaded onto suspension trapping (S-Trap) columns (Protifi, #C02-mini-80). S-Trap protocol was performed following the S-Trap manufacturer’s instructions. Trapped proteins were incubated with PNGase-F (New England BioLabs, NEB #P0704S) (20 units/μg of protein) at 37°C for 18h. PNGase F was previously diluted with 50 mM TEAB until a final volume of 25 μL. Tryptic (Promega, #V511A) (E:S, 1:50 w/w) digestion was conducted at 37°C for 16 h. The enzyme was diluted in 50 mM TEAB until a final volume of 100 μL. Tryptic peptides were dried in a vacuum concentrator (Martin Christ, Germany) and resuspended in formic acid 0.1%. Peptides were quantified using QuBit Assay (Thermo Scientific, #Q33212), following the manufacturer’s instructions.

#### Reverse phase nano-liquid chromatography tandem mass spectrometry and data analysis

Tryptic peptides (2 μg) were analyzed in an EASY-1000 nLC system (Thermo Scientific # LC120) coupled to a Q-Exactive Plus mass spectrometer (Thermo Scientific). The sample was loaded in a trap-column Acclaim PepMap 75 μm × 2 cm, nanoViper C18, 3 μm, 100 Å (Thermo Scientific, #164946) and separated in an analytical EASY-Spray Column PepMap column (75 μm × 25 cm, C18, 2 μm, 100 Å, Thermo Scientific, # 03-251-871). The mobile phases were 5% acetonitrile/0.1% formic acid (Solvent A) and 95% acetonitrile/0.1% formic acid (Solvent B). The gradient used was 10-45% B for 40 min, 45-70% B for 8 min, 70-95% for 5 min, and 95% for 7 min, at 300 nL/min of flow rate.

The mass spectrometry analysis employed the full-MS/DDA-MS2 and positive polarity modes. A dynamic exclusion list of 45 s and spray voltage at 1.9 kV was set for all studies. The parameter settings for the full scan were 1 microscan, 70,000 resolution at *m*/*z* 200, AGC target of 3E6 ions, 100 ms maximum injection time, and the range of mass acquired was *m*/*z* 350 to 2000 *m*/*z*. The top 20 DDA-MS2 parameters were 17,500 resolution, *m*/*z* 200, AGC target of 1E6 ions, 50 ms maximum injection time, *m*/*z* 2.0 of isolation window, minimum intensity threshold of 2E5 ions equipped with High-energy Collision Dissociation (HCD) cell using a normalized collision energy of 30 NCE.

The raw data were imported to Proteome Discoverer 2.4 (Thermofisher Scientific) software to perform protein identification analysis. A homemade database was created, including three sequences of spike variants (D614G, Gamma/P.1, and Delta/B.1.617.2). Additionally, a human database from UniProt (reviewed database with canonical and isoforms, June 2021) was used. Two searches were executed with total and semi-tryptic peptides, two missed cleavages were considered. Carbamidomethylation (C) was set as a fixed modification, whereas methionine oxidation, asparagine deamidation, and acetylation (protein N-terminal) were selected as variable modifications. The mass tolerance for precursor ions was 10 ppm and 0.1 Da for the fragment ions. The False Discovery Rate (FDR) was fixed for protein and peptide validation with a cutoff score minor to 1% at the protein, peptide and PSM levels. Proteins were grouped in master proteins using the maximum parsimony principle.

#### Analytic size-exclusion chromatography (A-SEC)

Spike variants at 25 μg/mL (injection volume: 250 μL) were directly injected into a Superose 6 increase 10/300 GL (Cytiva, #29091596). All runs were performed in PBS, PBS (140 mM NaCl, 2.8 mM KCl, 10 mM Na_2_HPO_4_, and 1.8 mM KH_2_PO_4_ pH 7.4) at 0.7 mL/min. Fluorescence was recorded at 350 nm upon excitation at 280 nm using a high-performance liquid chromatography system (Shimadzu Inc.). The column was calibrated using A_280nm_ with thyroglobulin (670 kDa), γ-globulin (158 kDa), ovalbumin (44 kDa), myoglobin (17 kDa), and vitamin B12 (1.35 kDa) (Bio-Rad, #151-1901). Column void was determined using blue dextran (2,000 kDa) at A_380nm_ and A_620nm_ (Merck, #D5751).

For trimer-to-monomer dissociation studies, a water bath (Fisher Scientific) at 40°C was kept close to the HPLC system, and samples were injected immediately after each time point (Figure 6). All HPLC runs were performed at room temperature. For guanidine experiments (Figure 4b), all runs were performed with the respective guanidine concentration (0.3 or 1 M).

#### Human ACE2 affinity

An indirect ELISA was performed to measure the affinity of the full-length ancestral, D614G, beta, gamma, and delta spike glycoprotein against the ACE-2 Fc protein. First, protein quantification was achieved by Pierce BCA Protein Assay Kit (Thermo Scientific, Cat# 23227, Batch# VH311372). 96-well plates were coated overnight at 4°C with 3-fold dilutions of each spike glycoprotein variant (tested concentrations between 50 μg/mL and 23 ng/mL). The 96-well plates were then washed with 0.05% PBS-Tween 20 (PBST) and blocked with 1% BSA in PBS (Sigma-Aldrich, Cat# A9647-50G, Lot# SLCH5268). Next, the plates were incubated with 2 μg/mL ACE-2 Fc protein (GenScript, Cat# Z03484-1, Lot# B2011010) at 37°C for 30 min and washed three times with 0.05% PBST. After that, anti-human IgG (Fc specific)-peroxidase antibody (Sigma-Aldrich, Cat# SAB3701282-2 MG, Lot# RI34900) was added at 1:10,000 dilution, and the plate was incubated at room temperature for 1 h. After rewashing the plate, TMB substrate (Life Technologies, Cat# 2023, Lot# 11323202-7) was added, and the reaction was stopped after 20 min at room temperature with HCl 1 N. The absorbance was read at 450 nm with 655 nm background compensation in iMark Microplate Absorbance Reader (BIO-RAD Laboratories). The half-maximal effective response (EC_50_) was carried out by fitting the curve to a four-parameter logistic regression by GraphPad Prism version 8 (GraphPad Software, San Diego, California, USA).

#### Circular dichroism (CD)

Far-UV spectra were recorded in a ChiraScan spectropolarimeter (Applied Photophysics) at wavelengths ranging from 200 to 260 nm at 25°C and 0.4 nm step size. Data is the average of five accumulation scans (Figure S5B). Raw ellipticities [θ] were recorded at a protein concentration of 50 μg/mL (final volume 300 μL) in a 2 mm path length cuvette using PBS. Data is shown as the mean residue ellipticity [Φ] and calculated as follows:

(Equation 1) $$\left[\Phi \right]\left(\deg.\frac{cm^{2}}{dmol}\right)=\frac{\left[\theta \right]\left(mdeg\right)}{10.L.c.n}$$

where *L* is the path length in centimeters, *c* is the molar concentration, and *n* is the number of peptide bonds (number of amino acids - 1).

#### Steady-state fluorescence spectroscopy

All experiments were performed using spike variants at 20 μg/mL. Fluorescence emission measurements were acquired using an ISSK2 spectrofluorometer (ISS Inc.) equipped with a high-pressure cell (ISS Inc.). Samples were excited at 280 nm to measure Tyr + Trp, and emission was recorded from 300 to 400 nm. Changes in the fluorescence spectra for each spike variant was quantified as the center of spectral mass (*ν*) (Equation 2), where *Fi* stands for the emitted fluorescence at wave number λ.

(Equation 2) $$\nu =\frac{\sum \lambda Fi}{\sum Fi}$$

From the center of spectral mass (*v*), we calculated the perturbation coefficient (α) as follows:

(Equation 3) $$\alpha =\frac{col\left(v\right)-v_{i}}{v_{f}-v_{i}}$$

Where *col(v)* stands for the center of spectral mass value in a specific guanidine concentration subtracted from its initial value (0 M guanidine, *vi*) and divided by the difference between the last (5 M guanidine, *vf*) and the first value.

We used 4,4′-dianilino-1,1′-binaphthyl-5,5′-disulfonic acid, bis-ANS to measure hydrophobic surface exposure (Invitrogen, #B153). A bis-ANS stock solution in PBS was prepared and quantified at A_385 nm_ using an extinction coefficient of 16,760 M^−1^ cm^−1^. To measure the bis-ANS fluorescence emission upon binding to spike variants, we mixed 2.4 μL of bis-ANS (stock at 5 mg/mL) with a range of 15–60 μL depending on the spike variant to have a final concentration of 40 μg/mL bis-ANS and 20 μg/mL of spike protein in 300 μL reaction volume (Figure 3D). Bis-ANS fluorescence was recorded from 400 to 600 nm upon excitation at 360 nm.

Guanidine (Merck, #607-148-00-0) titrations were carried out in the presence of 20 μg/mL of each spike variant. Samples were prepared individually for each guanidine concentration (0, 0.01, 0.02, 0.03, 0.05, 0.06, 0.07, 0.08, 0.1, 0.15, 0.2, 0.25, 0.3, 0.4, 0.5, 0.8, 0.9, 1, 1.3, 1.6, 1.9, 2.1, 2.5, 3, 3.5, 4, 4.5, and 5 M) and allowed to settle down at room temperature for 30 min before fluorescence acquisition. For these studies, the ancestral spike was either in PBS (pH 7.4) or 50 mM Tris-Cl containing 100 mM NaCl and 1 mM biotin at pH 8.0 (tris-based buffer). In this experiment, the ancestral protein, when tested in tris-based buffer, contained the TwinStrep and the 8xHis purification tags (Figure 4A), while all other analyses (ancestral and variant spike proteins in the presence of PBS) were carried out with samples of tagless spike proteins, which had been treated with HRV-3C protease (Figures 5A–5E). A non-linear mathematical fit was made according to the profile demonstrated by each experiment. Biphasic curves were adjusted using double Boltzmann equation and one-transition curves, using a Hill model built in OriginPro 8.1. G_50%_ values represent the mid-point for each transition.

Protein behavior as a function of pressure was investigated by applying pressure increments of 5,000 psi (∼344 bar) up to a maximum pressure of 3,500 bar. Upon each pressure increment, the pressure was allowed to stabilize for 5-min before bis-ANS fluorescence acquisition (Figure S9). For these experiments, we mixed 5.5 μL of bis-ANS (stock at 5 mg/mL) with a range of 15–60 μL depending on the spike variant to have a final concentration of 25 μg/mL bis-ANS and 25 μg/mL of spike protein in 1,100 μL reaction volume. For light scattering (LS) measurements, samples were excited at 320 nm, and the emission was recorded from 300 to 340 nm (Figure S9).

#### Dynamic light scattering (DLS)

Measurements were performed on a Brookhaven 90Plus/Bi-Mass multiple-angle particle sizing instrument. The autocorrelation curves C(τ) were fitted using multimodal size distribution (MSD), in which a non-negatively constrained least-square (NNLS) algorithm is used to produce an intensity or number-weighted distribution (Figure S7). Sample concentration was 50 μg/mL (final volume 100 μL) in PBS. Samples were allowed to rest for 20 min for thermal equilibration before acquisition at 25°C. C(τ) was acquired for 30 s for each run showing data as multiple replicates (Figure 3C).

### Quantification and statistical analysis

We used Minitab 17 software for statistical analysis. We performed a multiple comparison test for the half-maximal effective response (EC_50_), in which a one-way ANOVA was carried out, with a level of significance of 0.05. Model adequacy was examined by checking residual normality (Anderson-Darling test) and variance homogeneity (Bartlett test). The Post-Hoc Tukey test was implemented to determine which differences between pairs of EC_50_ means were significant.

## Data Availability

This study did not generate/analyze any datasets/code. Any additional information required to reanalyze the data reported in this paper is available from the lead contact upon request.

## Supplemental information

Document S1. Figures S1–S9

Document S2. Raw data source file
