# Supplementary material for: Conformational stability of SARS-CoV-2 glycoprotein spike variants
Source: iScience. 2022 Nov 30;26(1):105696. doi: 10.1016/j.isci.2022.105696 (PMC9710096; doi:10.1016/j.isci.2022.105696)
Supplement: Document S1. Figures S1–S9 [file mmc1.pdf]

iScience, Volume *26*

Supplemental information

Conformational stability
of SARS-CoV-2 glycoprotein spike variants

Hiam R.S. Arruda, Tulio M. Lima, Renata G.F. Alvim, Fernanda B.A. Victorio, Daniel P.B. Abreu, Federico F. Marsili, Karen D. Cruz, Mayra A. Marques, Patricia Sosa-Acosta, Mauricio Quinones-Vega, Jéssica de S. Guedes, Fábio C.S. Nogueira, Jerson L. Silva, Leda R. Castilho, and Guilherme A.P. de Oliveira

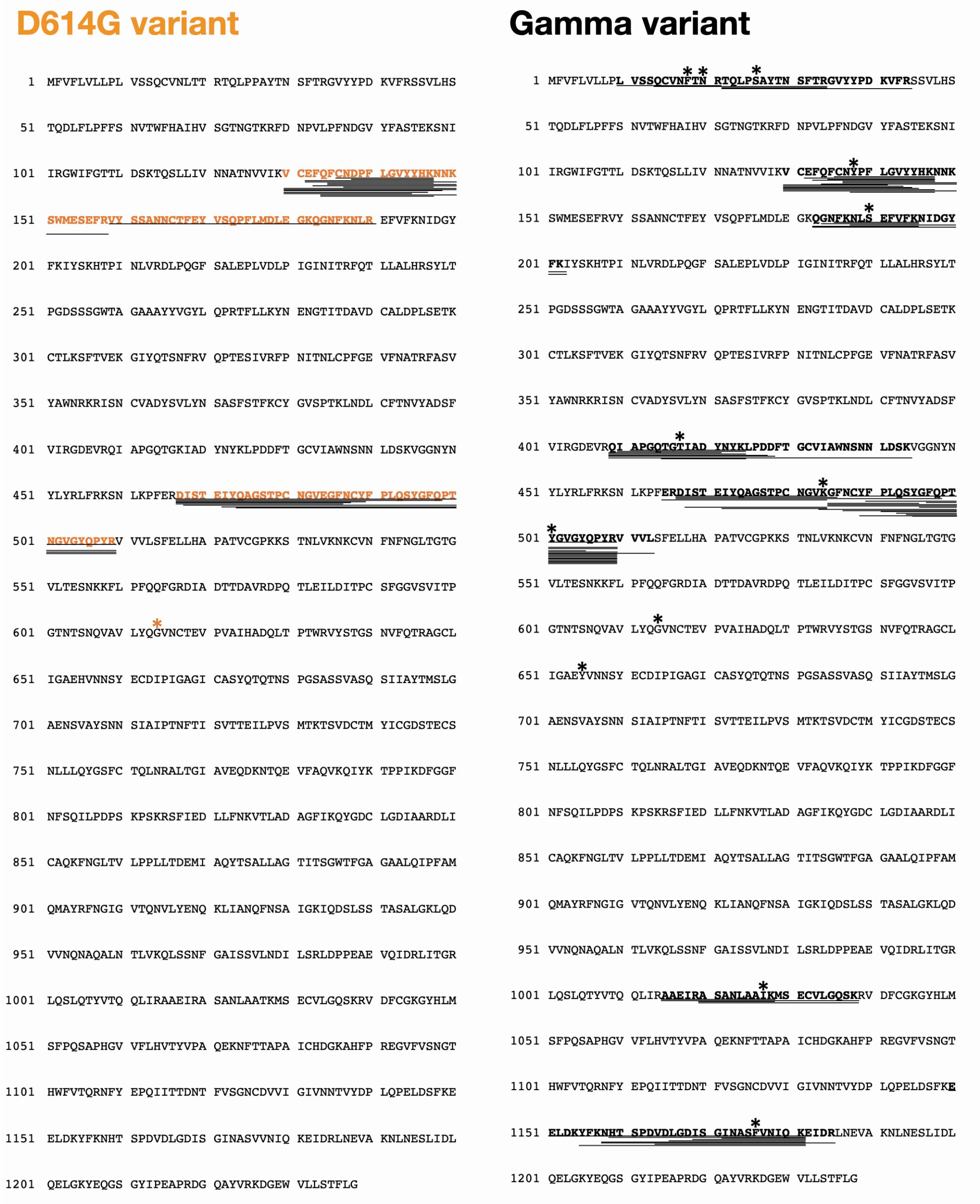

Figure S1. Related to figure 2. Distribution of unique peptides obtained on mass spectrometry runs of D614G and gamma variants after trypsin digestion. Identified peptides are underlined and bold colored. Colored asterisks depict altered sites of each variant. For a complete list of coverage peptides, check the source file appended.

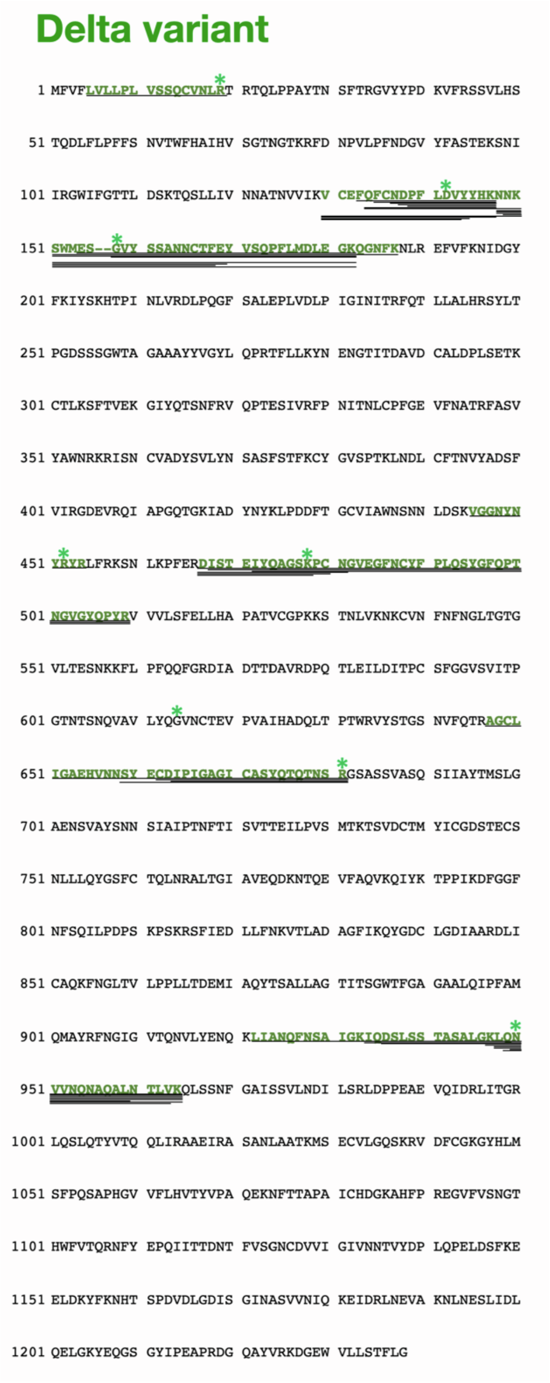

Figure S2. Related to figure 2. Distribution of unique peptides obtained on mass spectrometry runs of delta variant after trypsin digestion. Identified peptides are underlined and bold colored. Colored asterisks depict altered sites of each variant. For a complete list of coverage peptides, check the source file appended.

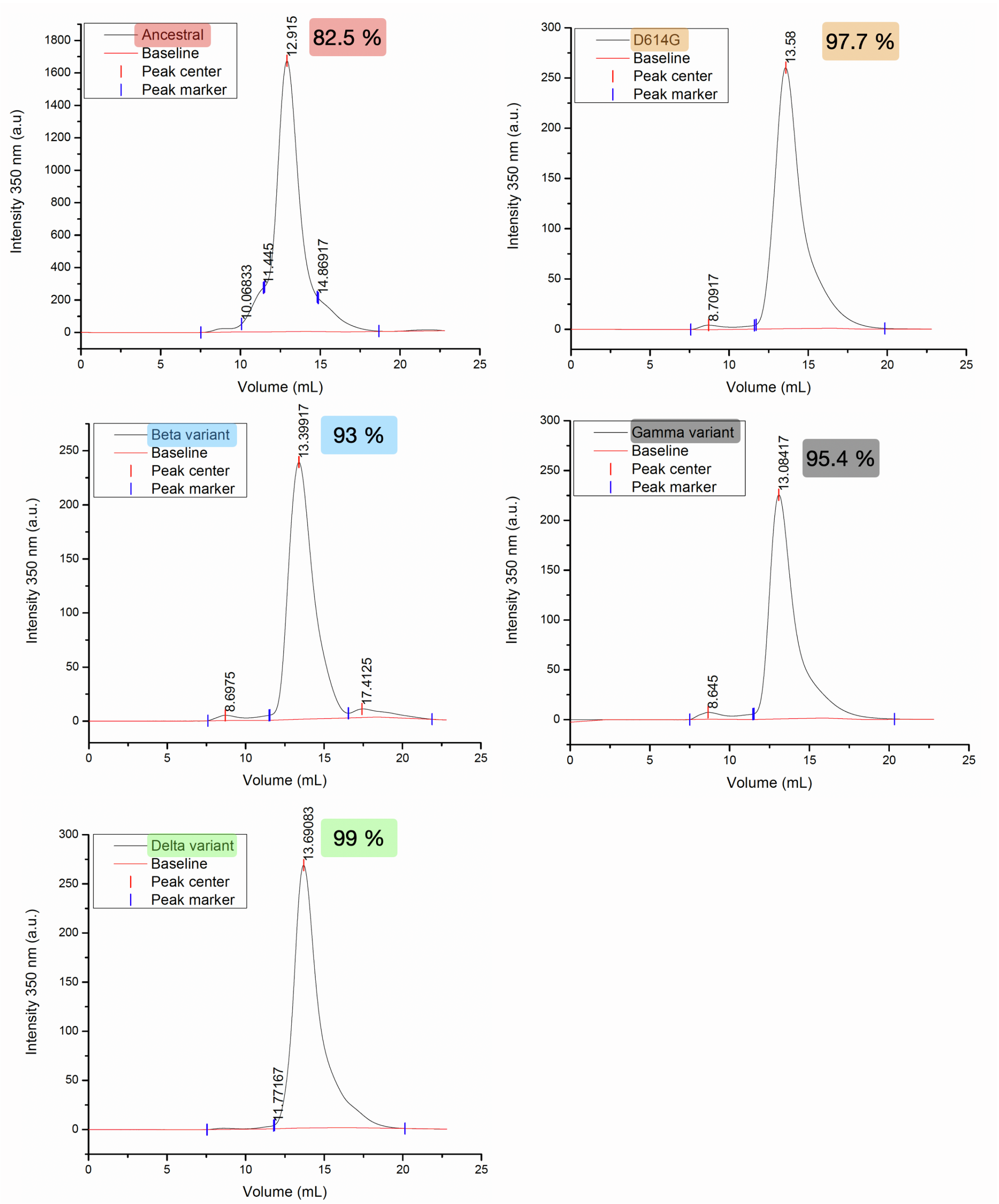

Figure S3. Related to figure 2. Quality checks of protein preparations. Peak integration analysis of SEC runs (Superose 6 10/300 GL) after protein preparation. Vertical values stand for retention volumes. Percentage area of corresponding spike peaks are color-coded.

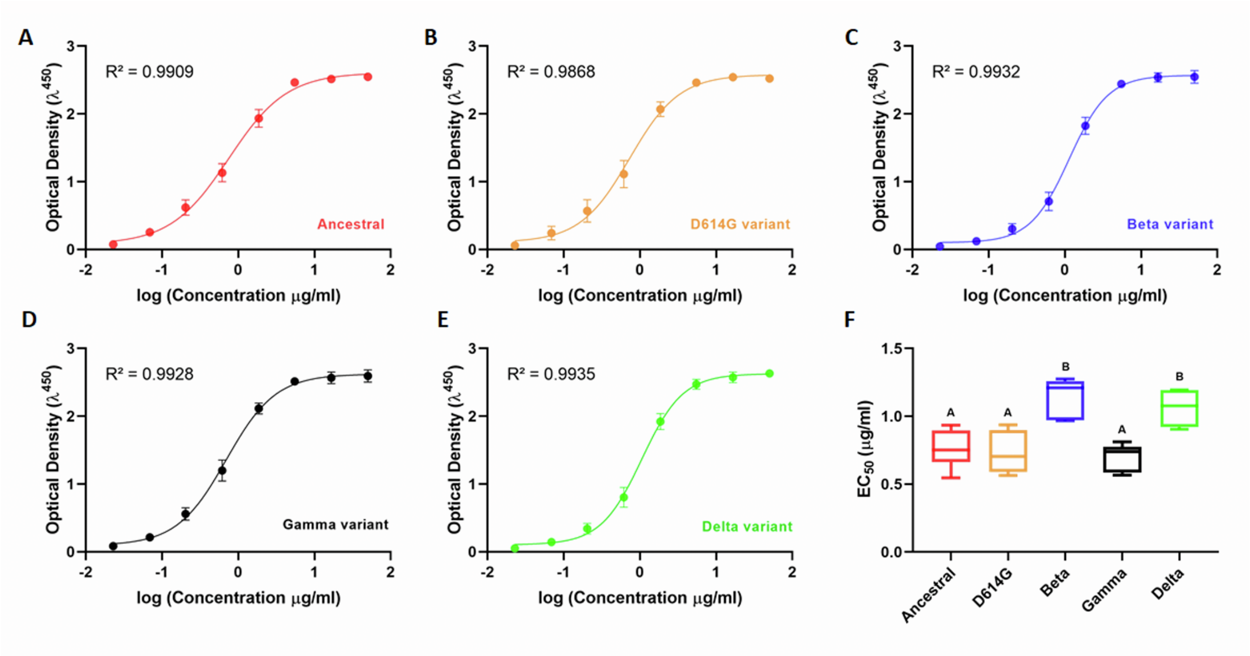

Figure S4. Related to figure 2. Binding assay on ACE2-Fc using different spike variants. Plots showing the optical density as a function of ACE2-Fc binding on coated wells with different spike glycoprotein concentrations (50 µg/ml to 23 ng/ml) for the **(A)** ancestral strain, **(B)** D614G, **(C)** beta, **(D)** gamma, and **(E)** delta variants. The data is shown as the avg. ± s.d. of (n = 6) repetitions. Each curve is plotted on a dose-response curve. **(F)** Half maximal effective concentration (EC_{50}) variation is represented on a Boxspot (median, upper, and lower quartiles, min, max). Means that do not share a letter are significantly different.

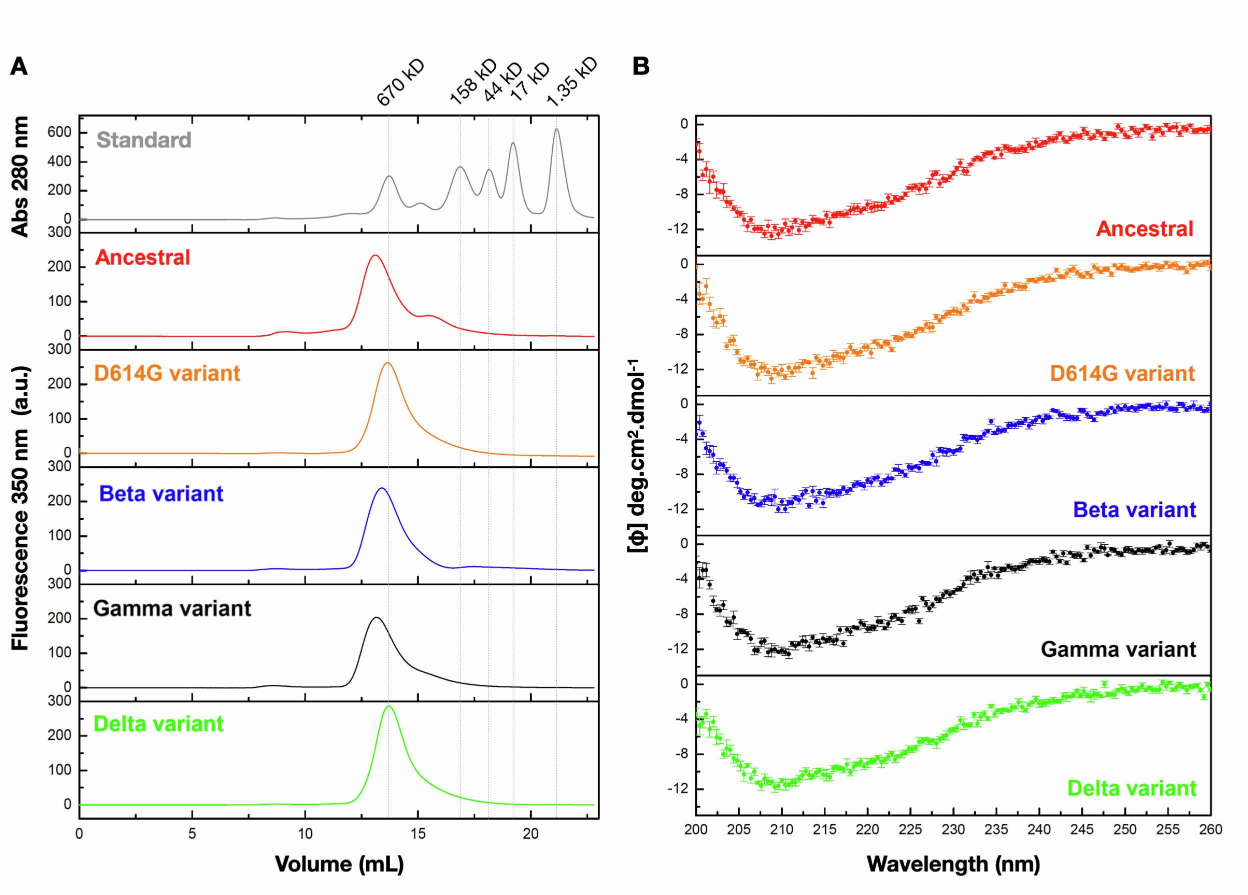

Figure S5. Related to figure 3.

(A) Line plots showing comparative SEC runs of the ancestral (red), D614G (orange), beta (blue), gamma (black), and delta (green) variants. Calibration ladder is colored gray. Vertical dashed lines show that spike variants eluted close to the 670 kDa peak with gentle differences for the ancestral and the gamma variants.

(B) Dot plots showing a collection of far-UV circular dichroism spectra of studied variants. [Φ] stands for the mean residue ellipticity (see methods). Data is shown as the avg. ± s.e.m (n = 5, replicates of the same batch). Color code is the same as in panel (A).

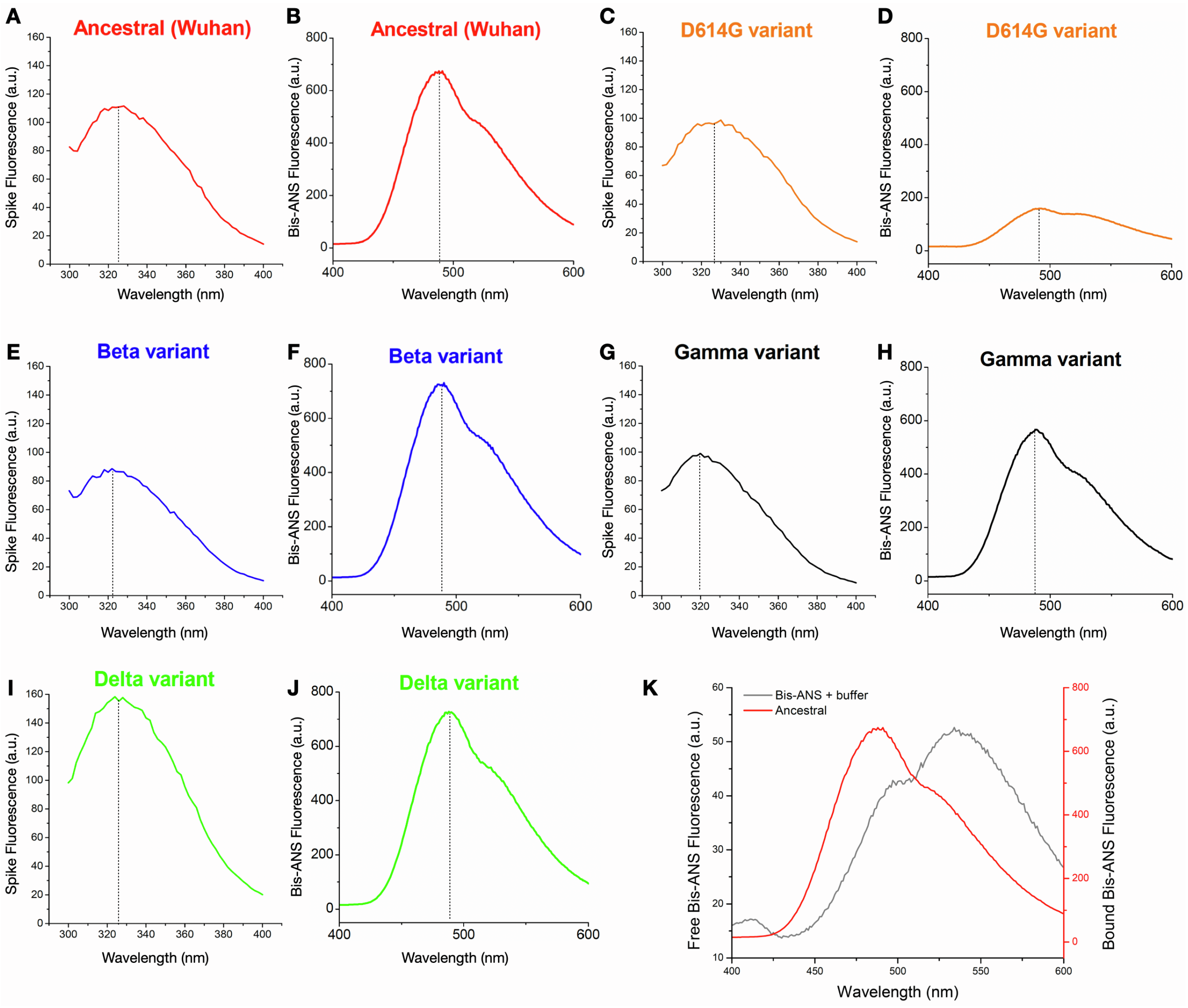

Figure S6. Related to figure 3. Line plots showing representative fluorescence emission of Trp residues upon excitation at 280 nm **(A, C, E, G, and I)** and of the bis-ANS probe upon excitation at 360 nm **(B, D, F, H, and J)** for the ancestral strain in red **(A, B)** and for D614G in orange **(C, D)**, beta in blue **(E, F)**, gamma in black **(G, H)**, and delta in green **(I, J)**. **(K)** Double-Y plot showing a control experiment of the bis-ANS fluorescence noise when free in solution (gray line) and bound to proteins (red line). A.u. stands for arbitrary units. Vertical dashed lines on panels (A-J) show maximum fluorescence emission (λ_{max}).

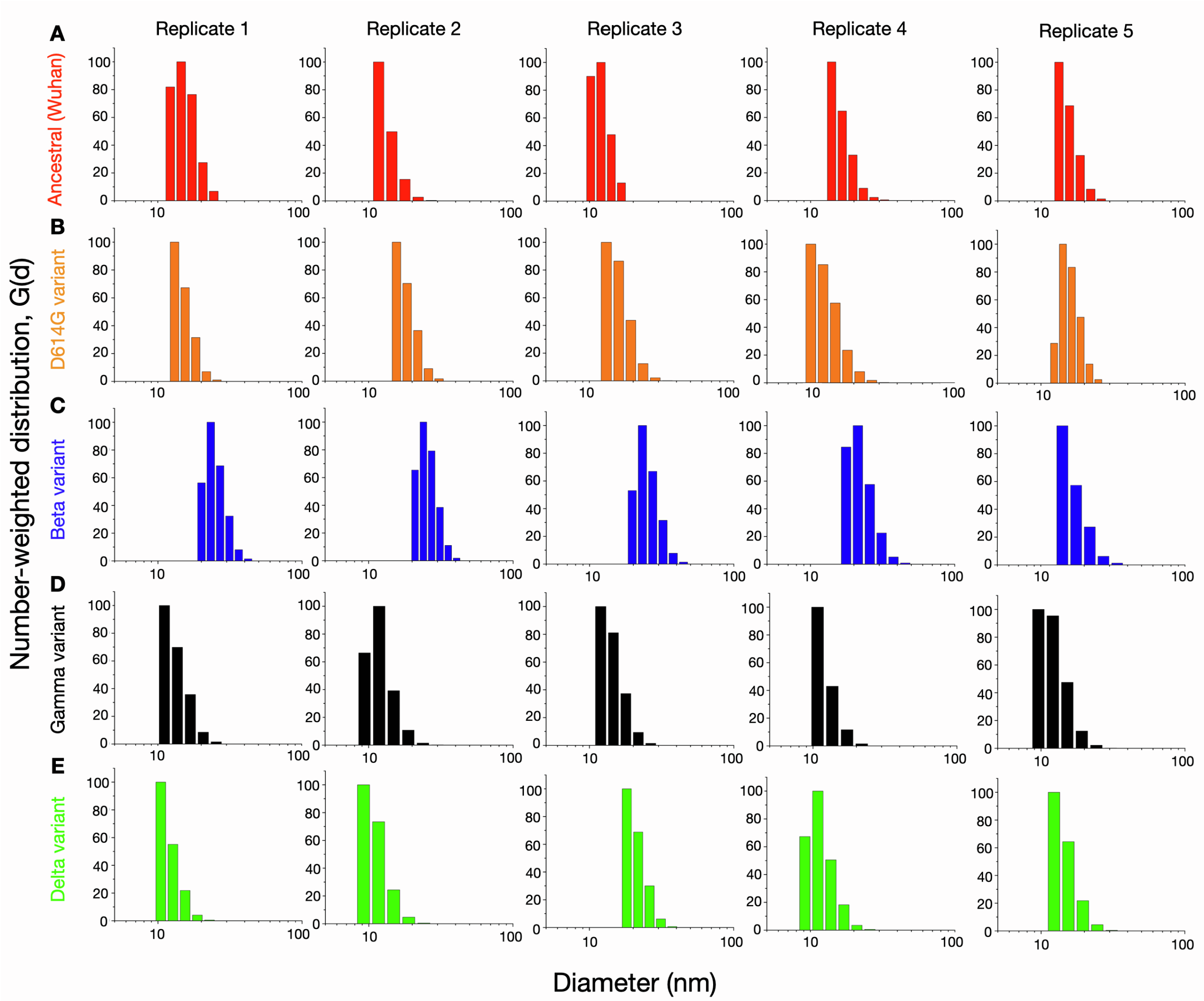

Figure S7. Related to figure 3. Dynamic light scattering (DLS) bar plots showing a collection of number-weighted distributions as a function of hydrodynamic diameter of **(A)** ancestral strain, **(B)** D614G, **(C)** beta, **(D)** gamma, and **(E)** delta variants. Data shows five replicates obtained from the same protein batch.

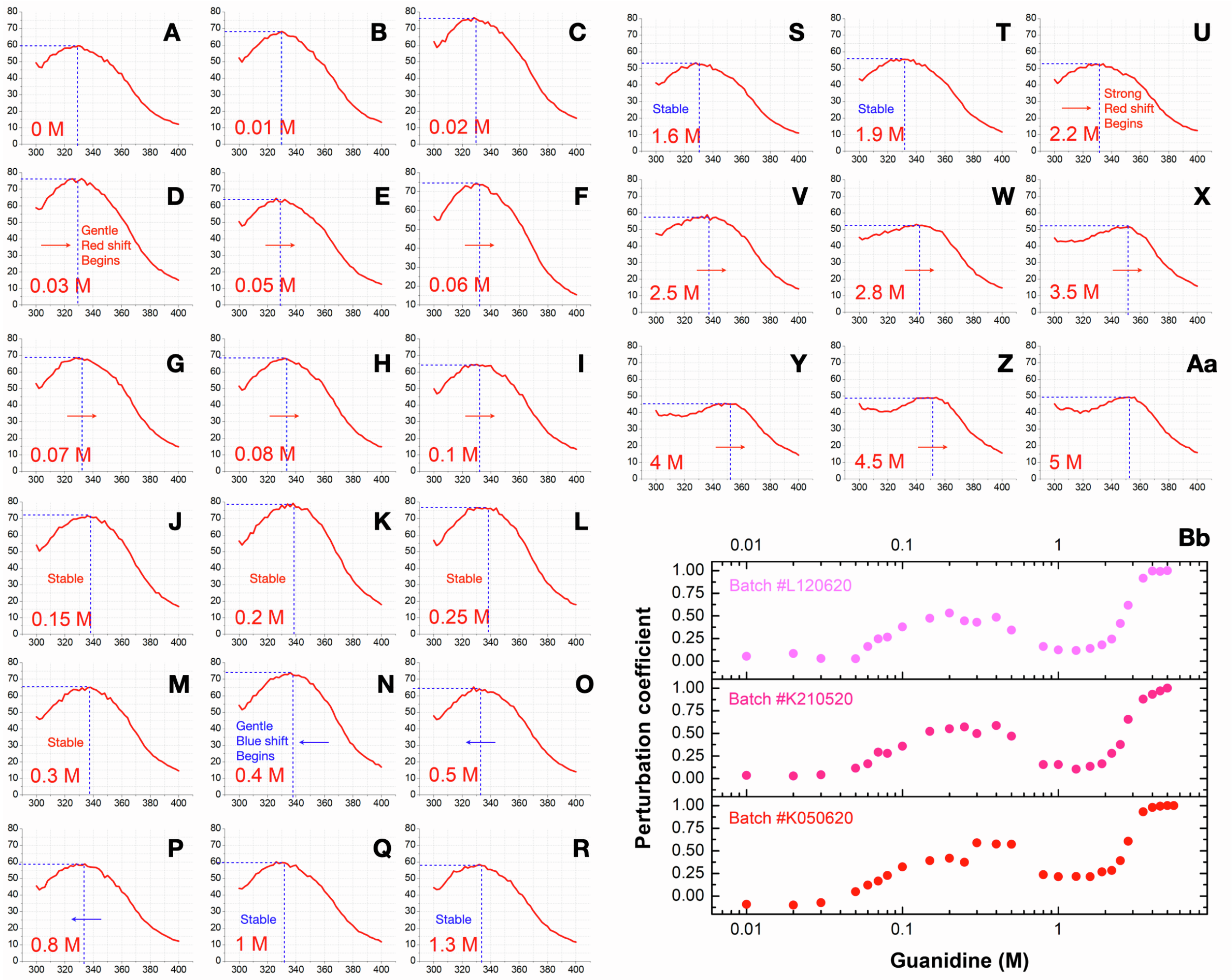

Figure S8. Related to figure 4. **(A-Aa)** Collection of line plots showing the fluorescence emission (y-axis) as a function of wavelength (x-axis) for the ancestral spike in the presence of the tris-based buffer (see Methods) and increasing concentrations of guanidine (labels in red, left bottom). During the chemical-induced unfolding of the spike, different regimens have been captured. **(A-C)** Gentle fluorescence increase (horizontal blue dashed lines); **(D-I)** Gentle redshift (red arrows); **(J-M)** Spectrum stability; **(N-P)** Gentle blueshift (blue arrows); **(Q-T)** Spectrum stability; **(U-Aa)** Strong redshift. Spectrum stability stands for the absence of appreciable intensity and red/blue shifts during guanidine increments. **(Bb)** Dot plots show the data consistency. Three independent protein preparations have shown the four-stage transition behavior.

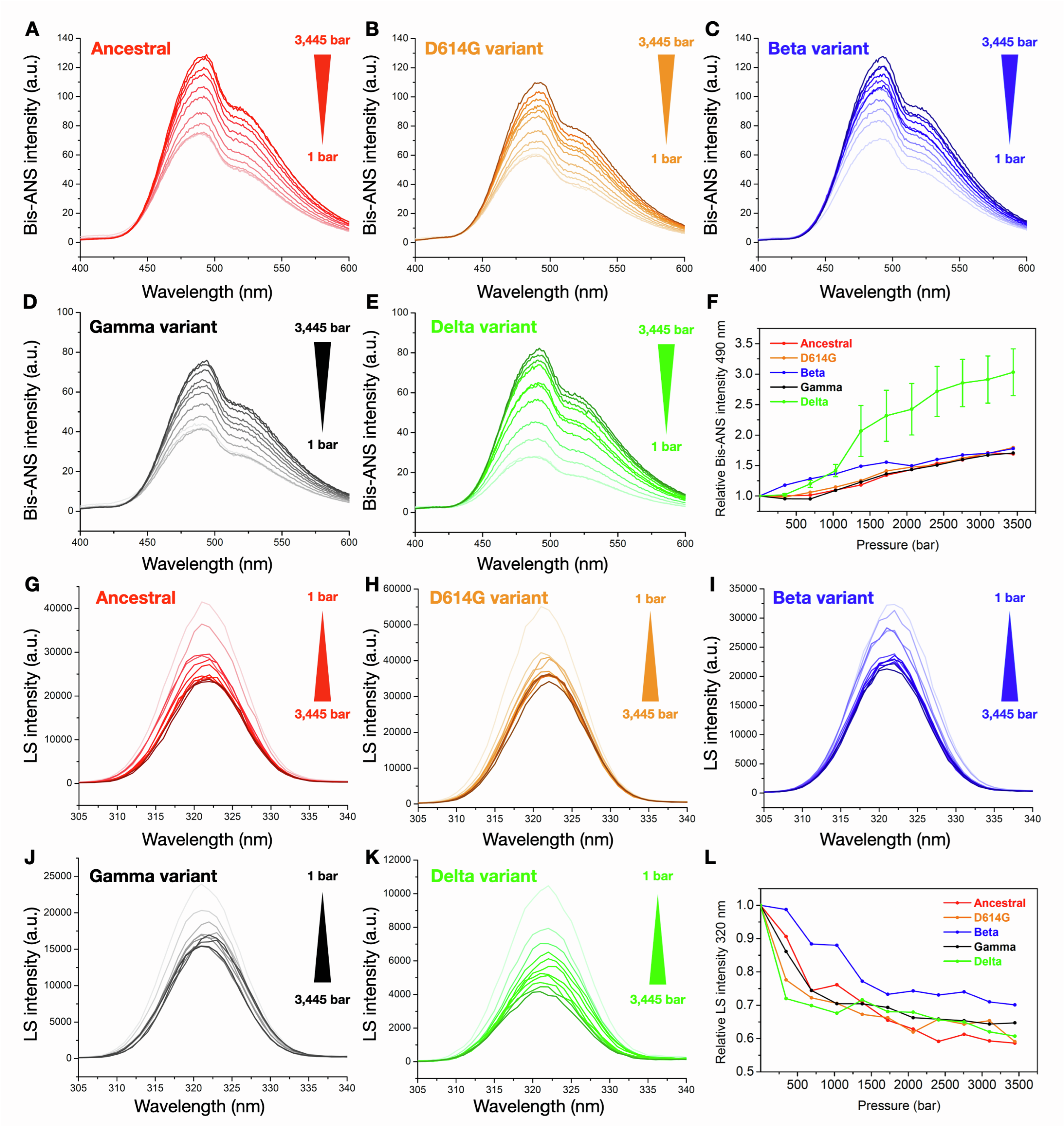

Figure S9. Related to figure 6. Line plots showing the response of **(A-E)** bis-ANS fluorescence emission and **(G-K)** light scattering (LS) to pressure increments from 1 up to 3,4 kbar for **(A, G)** ancestral strain, **(B, H)** D614G, **(C, I)** beta, **(D, J)** gamma, and **(E, K)** delta variants. Gradient color shows the spectra response to pressure increments (light color - lower pressure, dark color – higher pressure). **(F)** Relative bis-ANS fluorescence intensity and **(L)** light scattering intensity as a function of pressure increments for studied spikes. Data is shown as the avg. ± s.e.m. of independent experiments with three delta variant batches (n = 3). All other variants show n =1.
